# Engineering Mitochondrial Biogenesis in iPSC-CMs: CRISPR-Guided Approaches for Advanced Cardiomyocyte Development

**DOI:** 10.3390/jcdd13020077

**Published:** 2026-02-03

**Authors:** Dhienda C. Shahannaz, Tadahisa Sugiura, Brandon E. Ferrell, Taizo Yoshida

**Affiliations:** 1 Faculty of Medicine, Universitas Indonesia, Jakarta 16424, Indonesia; shahand@ccf.org; 2 Department of Cardiothoracic and Vascular Surgery, Montefiore Medical Center, Albert Einstein College of Medicine, Greene Medical Arts Pavilion 5th Floor, 3400 Bainbridge Avenue, New York, NY 10467, USA; 3 Department of General Surgery, Montefiore Medical Center, Albert Einstein College of Medicine, New York, NY 10467, USA

**Keywords:** mitochondrial biogenesis, iPSC-cardiomyocytes, CRISPR activation (CRISPRa), mitochondrial genome editing, PGC-1α signaling, cardiomyocyte maturation, oxidative phosphorylation (OXPHOS), mitochondrial dynamics, extracellular vesicle therapy, metabolic conditioning in iPSC-CMs

## Abstract

Human iPSC-derived cardiomyocytes (iPSC-CMs) exhibit fetal-like mitochondrial networks and limited oxidative metabolism, constraining their translational utility. The key bottleneck is mitochondrial immaturity, resulting from blunted PGC-1α–*NRF1/2–TFAM* axis activation and insufficient nuclear–mitochondrial coordination, rather than sarcomeric or electrophysiological immaturity alone. This review synthesizes genome-guided interventions (CRISPRa and mtDNA editing) and complementary environmental strategies—including metabolic substrate switching, electromechanical stimulation, and extracellular vesicle (EV)-mediated mitochondrial transfer—to drive mitochondrial biogenesis and maturation in iPSC-CMs. We systematically reviewed studies (2005–2025) targeting (1) key regulators of mitochondrial biogenesis (PGC-1α, *NRF1/2*, *TFAM*), (2) CRISPR-based transcriptional activators/repressors and mtDNA editors (DdCBE, mitoTALENs), and (3) maturation approaches such as metabolic conditioning, electromechanical stimulation, 3D tissue culture, and EV-mediated mitochondrial transfer. CRISPRa-mediated activation of PGC-1α, *NRF1*, and *GATA4*, combined with mtDNA base editors, enhances mitochondrial mass and OXPHOS function, while integration with environmental maturation strategies further promotes adult-like phenotypes. Integrative approaches that combine genome-guided interventions (CRISPRa, mtDNA editing) with environmental maturation cues yield the most adult-like iPSC-CM phenotypes reported to date. CRISPR-guided mitochondrial biogenesis thus represents a frontier for producing metabolically competent, structurally mature iPSC-CMs for disease modeling and therapy. Remaining translational challenges include efficient mitochondrial delivery, metabolic homeostasis, and multi-omics validation. We propose standardized workflows to couple nuclear and mitochondrial editing with maturation strategies.

## 1. Introduction

Human-induced pluripotent stem-cell-derived cardiomyocytes (iPSC-CMs) have emerged as a transformative platform for cardiac disease modeling, cardiotoxicity screening, and regenerative therapy development [1]. Despite significant progress, their translational application remains limited by morphological, metabolic, and electrophysiological features that resemble fetal rather than adult cardiomyocytes [1,2,3,4,5,6,7]. These deficiencies are particularly pronounced in mitochondrial form and function [4], which impose key bottlenecks for both disease modeling and therapeutic applications [4]. Limited mitochondrial mass, fragmented cristae, and impaired calcium handling collectively undermine the fidelity and functional potential of iPSC-CMs [4]. The clinical need is thus clear: strategies that induce adult-like mitochondrial competency are critical to generate cardiomyocytes capable of modeling disease accurately and supporting myocardial repair.

Mitochondrial maturation is central to adult cardiomyocyte function. Mature mitochondria are densely networked, optimized for fatty-acid oxidation, and responsible for ~70–90% of ATP production [4]. They maintain calcium homeostasis, control reactive oxygen species (ROS), and support excitation–contraction coupling [4]. In iPSC-CMs, immature mitochondria lead to inadequate ATP supply, impaired Ca^2+^ handling, and heightened oxidative stress [2,3,4]. Functional deficits in excitation–contraction coupling, bioenergetics, and stress resilience limit translational reliability, highlighting mitochondrial biogenesis as a critical target for intervention [4].

To overcome this barrier, there is growing interest in engineering mitochondrial biogenesis—the process by which new mitochondria are generated and integrated into cellular networks—as a targeted maturation strategy [4,5,6]. Recent studies have explored metabolic conditioning, mechanical/electrical stimulation, 3D engineered-tissue culture, and extracellular vesicle (EV)-mediated mitochondrial transfer [7,8,9]. While these approaches yield partial improvements in mitochondrial content and OXPHOS function, full adult-level mitochondrial maturation remains elusive [4]. For example, fatty-acid supplementation or electromechanical training increases mitochondrial number but yields only partial functional improvement [4]. While these strategies advance maturation, their efficacy remains incomplete and variable across studies. Among existing maturation strategies, environmental and metabolic interventions predominantly enhance mitochondrial quantity. However, they rarely reconstitute the transcriptional and genomic coordination required for durable adult-like bioenergetics. By contrast, genome-guided modulation of mitochondrial regulators uniquely targets the upstream control nodes governing mitochondrial identity, positioning it as a higher-leverage strategy [4]. Crucially, modulation of these upstream regulators directly translates into downstream functional gains—enhancing oxidative phosphorylation capacity, calcium buffering, and excitation–contraction coupling—rather than merely increasing mitochondrial abundance.

Emerging genome-engineering approaches, particularly CRISPR-guided tools, provide a promising solution. CRISPRa-mediated transcriptional activation of master regulators—PGC-1α, *NRF1/2*, and *TFAM*—can program mitochondrial network expansion and OXPHOS machinery assembly. Mitochondrial genome editors, such as DdCBE and mitoTALENs, enable targeted mtDNA sequence modification. Yet, application of these tools specifically to enhance mitochondrial maturation in iPSC-CMs remains sparse. Importantly, genetic editing strategies are rarely integrated with environmental maturation cues, such as metabolic conditioning, electromechanical stimulation, or EV-mediated delivery [10,11,12,13,14,15].

This represents a clear novelty gap in current research [10,11,12,13,14,15]:

- Few studies combine CRISPRa-mediated nuclear activation with mtDNA editing in iPSC-CMs to synergistically enhance mitochondrial biogenesis.
- Functional validation of edited mitochondria (ATP production, ROS handling, calcium buffering, excitation–contraction coupling) is rarely comprehensive.
- Delivery strategies for CRISPRa or mitochondrial base editors remain inefficient, cytotoxicity-prone, and under-optimized.
- Integration with environmental cues (mechanical/electrical stimulation, metabolic conditioning, 3D culture, EV delivery) is underexplored.
- Timing, dosage, and stability of editing interventions are poorly defined.
- Long-term translational validation in engineered heart tissues or in vivo transplantation is limited.
- Multi-omics confirmation of adult-like mitochondrial identity is often missing.
- Variability among iPSC lines and cardiomyocyte subtypes (atrial, ventricular, nodal) is largely unaddressed.
- Correction of disease-relevant mtDNA variants and functional rescue strategies are nascent.
- Standardization of tools and protocols for reproducibility is lacking.

**Figure 1:**
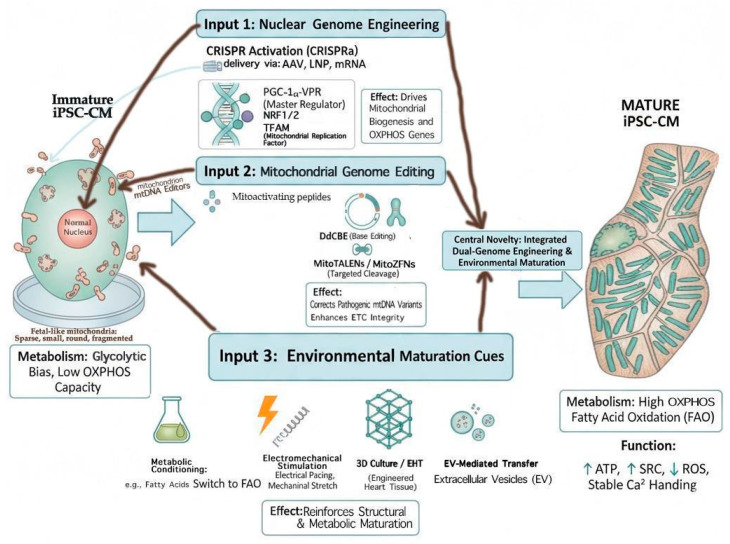
Overview schematic of CRISPRa-driven nuclear activation, mtDNA editing, and environmental maturation approaches in iPSC-CMs. This diagram highlights the central novelty of integrating nuclear and mitochondrial genome engineering with metabolic, electromechanical, and EV-mediated cues to drive adult-like cardiomyocyte mitochondrial maturation. The brown arrows originating from Input 1 (Nuclear Genome Engineering) and Input 2 (Mitochondrial Genome Editing) represent targeted genetic interventions. Arrows to the Cell: These arrows point back to the “Immature iPSC-CM,” indicating the delivery of CRISPRa machinery (via AAV or mRNA) and mtDNA editors (like DdCBE or MitoTALENs) into the cell’s nucleus and mitochondria. 1. Arrows to the Central Novelty: They also flow toward the center, signifying that these two genetic strategies are being combined—rather than used in isolation—to create a synergistic effect on the cell’s biology. 2. Environmental & Metabolic Cues: The Input 3 (Environmental Maturation Cues) section uses arrows to show the application of external stimuli to the developing tissue. Feedback to the Cell: The upward-pointing arrow directed at the immature cell indicates that physical and chemical “conditioning” (like fatty acid switches and electrical pacing) pushes the cell away from its “fetal-like” state, To the Central Novelty: Like the genetic inputs, this arrow feeds into the “Integrated Dual-Genome Engineering” block, showing that environmental cues are the third essential pillar of this maturation protocol. 3. The Path to Maturation The large light-blue horizontal arrows represent the chronological progression of the cell over time. The Transition: They track the shift from the Immature iPSC-CM (left)—characterized by a glycolytic metabolism and fragmented mitochondria—through the “Integrated” processing stage, and finally toward the Mature iPSC-CM (right). The Outcome: The final arrow points to the elongated, highly organized mature cell, which now boasts high OXPHOS capacity, stable calcium handling, and increased ATP production.

To provide a visual summary of our integrative approach, we present Figure 1, which schematically unifies CRISPRa-mediated nuclear activation, mitochondrial genome editing (DdCBE/mitoTALENs), and complementary maturation strategies.

To address these gaps, this review systematically synthesizes strategies for engineering mitochondrial biogenesis in iPSC-CMs, emphasizing CRISPR-guided nuclear and mitochondrial interventions. We contextualize these tools alongside metabolic, mechanical, 3D tissue, and EV-based approaches, providing an integrative roadmap for advanced cardiomyocyte development. Coverage spans research and review from 2005–2025, highlighting: (1) regulators of mitochondrial biogenesis (PGC-1α, *NRF1/2*, *TFAM*), (2) CRISPR-based transcriptional modulators and mitochondrial genome editors, and (3) combinatorial maturation strategies. Emphasis is placed on studies using human iPSC-CMs, engineered tissues, and in vivo translation experiments to maximize clinical relevance.

**Figure 2:**
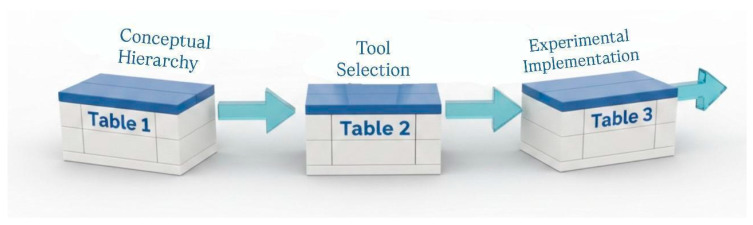
Integrative schematic linking mitochondrial maturation tables. This figure illustrates the logical flow of the review: Table 1 summarizes the conceptual hierarchy of mitochondrial interventions (Tier I–III), Table 2 details tool-specific functional outcomes, Table 3 summarizes environmental cues, and Table 4 outlines recommended experimental workflows. Arrows indicate the progression from conceptual understanding → tool selection → experimental implementation.

By bridging genome editing with environmental and EV-mediated cues, we propose pathways to produce metabolically and structurally mature iPSC-CMs capable of adult-level function. Our review identifies translational barriers—including safe delivery, metabolic homeostasis, multi-omics validation, and reproducibility—and proposes standardized experimental pipelines and best-practice frameworks. This comprehensive synthesis aims to catalyze next-generation strategies for disease modeling and regenerative cardiology, positioning CRISPR-guided mitochondrial biogenesis as a cornerstone of iPSC-CM maturation. As summarized in Table 1, maturation strategies can be ordered by their degree of control over mitochondrial identity, from upstream transcriptional and genomic regulation to downstream environmental and biophysical modulation, discussed in Section 5, Section 6 and Section 7. To clarify why certain approaches consistently yield partial maturation, we organize existing strategies by their position within the mitochondrial control hierarchy rather than by experimental modality. The logical progression from this conceptual hierarchy to tool selection and experimental implementation is illustrated in Figure 2.

**Table 1:** Current Limitations & Future Directions [9,10,11,12,13].

Category	Current Limitations	Future Directions/Unmet Needs
Tier I—Identity-setting interventions (Upstream Control) Define mitochondrial identity, biogenesis capacity, and genome integrity		
Nuclear Editing (CRISPRa/CRISPRi)	Limited application to core regulators (PGC-1α, *NRF1/2*, *TFAM*); inconsistent transcriptional upregulation	Systematic evaluation of dose, timing, and combinatorial activation; integration with downstream environmental maturation strategies (metabolic, electromechanical, EVs) to achieve adult-like mitochondrial identity.
Mitochondrial Genome Editing (DdCBE, mitoTALENs)	Proof-of-concept studies; inefficient delivery; off-target effects; heteroplasmy control challenges	Optimized mtDNA-targeting vectors; durable heteroplasmy shifts; long-term stability; multi-omics validation; maximal benefit achieved when paired with nuclear activation and environmental conditioning.
Tier II—Network Coordination Interventions (Mid-Level Control) Align mitochondrial expansion with metabolic demand, structure, and quality control		
Metabolic Conditioning (FAO induction, substrate switching)	Partial maturation; persistent glycolytic bias; variable reproducibility	Standardized substrate-switching protocols; synergistic with nuclear/mtDNA editing; evaluate FAO flux and reserve capacity in integrated maturation platforms.
Electrical/Mechanical Stimulation	Modest increases in mitochondrial content; limited functional translation	Define optimal waveform, frequency, and duration; effective only when combined with genome-guided interventions for synchronized energetic and contractile maturation.
Extracellular Vesicle (EV) Delivery/Mitochondrial Transfer	Isolated reports; limited scalability; inconsistent mitochondrial incorporation	Scalable, reproducible EV production; enhances OXPHOS and network connectivity most effectively when paired with Tier I editing strategies.
Tier III—Functional Validation & Translational Reinforcement (Downstream Control) Assess, stabilize, and translate mitochondrial maturation		
Functional Validation	Reliance on single readouts (ATP, ROS); limited systems-level assessment	Integrated multi-omics (transcriptomics, proteomics, metabolomics, epigenomics), excitation–contraction coupling, and Ca^2+^ imaging; full phenotypic maturation is achieved only when combined with Tier I genome-guided interventions and Tier II environmental strategies (FAO, electromechanics, EV transfer).
Translational/In Vivo Relevance	Limited in vivo testing; immature phenotype persists post-transplant	Preclinical studies with engineered tissues; long-term functional integration and adult-like mitochondrial phenotype depend on synergistic Tier I + II interventions, including nuclear/mtDNA editing and metabolic/mechanical conditioning.
Line/Subtype Variability	Inconsistent maturation across iPSC lines and cardiac subtypes	Cell-line-specific optimization; tailored strategies for atrial, ventricular, and nodal cardiomyocytes; ensuring effective integration of genome-guided and environmental maturation approaches for consistent adult-like function.
Standardization & Reproducibility	Variability in tools, assays, and culture conditions	Establish best-practice frameworks, reporting standards, and reproducible pipelines; protocols should incorporate integrated genome-guided and environmental interventions, coupled with multi-level functional readouts, to ensure consistent maturation across labs.

Strategies are ordered by level of control over mitochondrial identity, progressing from upstream transcriptional and genomic regulation to downstream environmental and biophysical modulation. Integration is essential: Tier II and III strategies achieve adult-like phenotypes only when paired with Tier I genome-guided interventions. Functional metabolic readouts for each intervention are summarized in Table 2.

In aggregate, the central limitation restraining the translational utility of iPSC-derived cardiomyocytes is their persistent mitochondrial immaturity, encompassing insufficient mitochondrial mass, incomplete cristae architecture, suboptimal oxidative metabolism, and impaired excitation–contraction coupling. These deficits constrain disease modeling fidelity, cardiotoxicity prediction, and regenerative efficacy. Consequently, engineering adult-like mitochondrial structure and function—rather than sarcomeric or electrophysiological maturation alone—emerges as a foundational requirement for clinically and mechanistically relevant iPSC-CMs. This review is therefore anchored on the premise that targeted mitochondrial biogenesis, particularly through integrative CRISPR-guided nuclear and mitochondrial interventions combined with physiologic maturation cues, represents a decisive strategy to overcome this bottleneck.

In summary, mitochondrial immaturity underlies functional limitations in iPSC-CMs. Upstream genome-guided interventions (CRISPRa, mtDNA editing) provide high-leverage control over organelle identity, while environmental cues reinforce network maturation. These combined strategies form the foundation for targeted engineering of adult-like cardiomyocytes, as detailed in the following sections.

## 2. Materials and Methods

### 2.1. Literature Search Strategy

A comprehensive literature search was performed in PubMed, Google Scholar, Scopus, Web of Science, bioRxiv/medRxiv, and the MDPI database covering 2005–2025. Search terms included: “iPSC-cardiomyocytes,” “mitochondrial biogenesis,” “PGC-1α *NRF1 TFAM*,” “CRISPRa,” “mitochondrial DNA editing,” “DdCBE,” “mitoTALEN,” “cardiomyocyte maturation,” “extracellular vesicle mitochondrial transfer,” and “engineered heart tissue maturation”.

### 2.2. Eligibility Criteria

We included primary research and reviews involving:

- Mitochondrial biogenesis, dynamics, or metabolic maturation in iPSC-CMs;
- Nuclear–mitochondrial regulatory pathways (PGC-1α, *ERRα*, *NRF1/2, TFAM*);
- CRISPRa/CRISPRi for metabolic or mitochondrial gene modulation;
- mtDNA editing platforms (DdCBE, mitoTALEN, TALED);
- Environmental maturation strategies (metabolic substrate switching, mechanical/electrical stimulation, 3D tissues);
- Extracellular vesicle-mediated mitochondrial transfer;
- Preclinical in vivo studies using iPSC-CMs or engineered cardiac tissues;
- The author’s published work on iPSC therapy, mitochondrial EVs, energetics restoration, arrhythmogenicity, and iPSC-CM maturation.

Exclusion Criteria

We excluded:

- Non-cardiac cell studies without transferable mitochondrial mechanisms;
- Non-CRISPR gene engineering (unless comparator);
- Studies lacking functional mitochondrial assays;
- Duplicates, commentaries, and conference abstracts.

### 2.3. Study Screening and Selection

Two reviewers independently screened titles/abstracts and evaluated full texts. Disagreements were resolved by consensus. A PRISMA-style approach was followed to document: records identified, screened, assessed, and included.

### 2.4. Data Extraction

To capture both nuclear and mitochondrial determinants of iPSC-CM maturation, we extracted parameters across editing modalities, environmental interventions, and functional readouts. For each eligible study, the following parameters were extracted:

1. Mitochondrial Biogenesis
  - PGC-1α/β, *NRF1/2, ERRα, TFAM* expression levels;
  - mtDNA copy number;
  - Mitochondrial mass and morphology;
  - Cristae density (EM);
  - OXPHOS complex activity;
  - OCR/ECAR profiles.
2. CRISPR-Based Manipulation
  - CRISPRa system (dCas9-VPR, dCas9-SAM, dCas9-p300);
  - gRNA design strategy and target loci;
  - Editing efficiency and off-target analysis;
  - Delivery modality (viral, non-viral, EV/nanoparticles).
3. Mitochondrial DNA Editing Tools
  - DdCBE/mitoBEs used (cytidine/adenosine);
  - On-target/off-target mtDNA change;
  - Sequencing methodology (NGS, long-read, duplex sequencing).
4. Maturation Interventions
  - Metabolic substrate switching (FA, galactose, lactate deprivation);
  - Electrical stimulation (Hz, waveform);
  - Mechanical loading/3D engineered tissues;
  - EV-mediated mitochondrial rescue (source, cargo characterization).
5. Functional Readouts
  - Sarcomere maturation;
  - APD90 (action potential duration at 90% repolarization; prolonged APD90 indicates immaturity or arrhythmogenicity, whereas shortened APD90 reflects enhanced electrophysiological maturation), detailed AP shape parameters (dV/dtmax, Vmax), and comprehensive Ca2+ transient kinetics (rise time, decay τ, amplitude, reuptake rate via SERCA and NCX profiling);
  - ROS homeostasis;
  - Arrhythmogenicity indices;
  - Contractile stress (force generation in EHTs).

For each eligible study, we recorded not only experimental parameters but also whether functional and metabolic readouts were reported. Metrics included mtDNA copy number, OCR (basal and maximal), spare respiratory capacity, ROS production, calcium transient fidelity, and sarcomere ultrastructure. This allowed a comparative assessment of the comprehensiveness and reproducibility of maturation studies and identification of gaps that CRISPR-guided interventions could address. In summary, the extracted parameters capture the full spectrum of determinants shaping iPSC-CM maturation, spanning nuclear, mitochondrial, and environmental influences. By linking genetic interventions with functional readouts, this dataset enables comparative evaluation of strategies and highlights translational gaps. The categories encompass both upstream regulators (nuclear and mtDNA) and downstream outcomes (metabolic and electrophysiological), providing an integrated framework to assess combinatorial approaches for achieving adult-like cardiomyocyte function.

### 2.5. Sequencing Methodology and Variant Interpretation

To ensure rigorous evaluation of mitochondrial genome editing outcomes, we extracted detailed sequencing-related parameters from all included studies. Extracted variables included: (1) sequencing platform (short-read NGS, long-read, or duplex sequencing), (2) average and per-base coverage depth, (3) error-correction strategies, and (4) variant-calling thresholds relevant to heteroplasmy quantification.

Short-read NGS datasets were assessed for read quality (Q ≥ 30) and adapter trimming. Alignment parameters were optimized for mtDNA circularity. Minimum accepted mean coverage was ≥500×, with ≥1000× used as the preferred threshold for detecting low-frequency heteroplasmy (<1%).

Long-read sequencing (PacBio HiFi, ONT) was extracted when studies evaluated large mtDNA deletions, structural variants, or mtDNA-nuclear DNA chimeric reads. For long-read platforms, high-fidelity error filtering or consensus-building algorithms (e.g., R10.4.1 Q20+, circular consensus sequencing) were documented.

Duplex sequencing datasets were evaluated separately due to their ultra-low error rate (≈10^−7^–10^−8^). Studies using duplex consensus sequencing (DCS) were annotated for strand-tagging methodology, unique molecular identifiers (UMIs), and the required ≥500× DCS depth to confirm low-frequency off-target edits.

Variant interpretation parameters included: (1) heteroplasmy detection limits (0.1%, 0.5%, or 1%), (2) allele-fraction thresholds for calling on-target edits (≥5% unless justified), and (3) filters for strand bias, read position bias, and background error models. Studies lacking sufficient coverage or appropriate mitochondrial-specific variant filters were flagged during quality assessment.

Because mtDNA is present in high copy number, we extracted reporting on copy-number normalization strategies (e.g., nuclear DNA ratios, *TFAM*-corrected quantification) to ensure accurate interpretation of variant allele fractions in edited iPSC-CMs.

CRISPRa activation of PGC-1α is prioritized for its established role in increasing mitochondrial mass and OXPHOS activity. DdCBE was selected due to its precise cytosine editing and low off-target potential in the mitochondrial genome. PGC-1α/NRF1 transcriptional activation is linked to increased mtDNA copy number, cristae density, and OXPHOS activity, demonstrating functional maturation. EV-mediated mitochondrial transfer complements these edits by improving ROS homeostasis and calcium handling. Together, these parameters allowed cross-study normalization of mtDNA editing efficiency, off-target assessment, and heteroplasmy dynamics in iPSC-CM maturation paradigms.

### 2.6. Data Synthesis

A narrative synthesis approach was used to integrate nuclear–mitochondrial pathways with mtDNA maintenance mechanisms, group CRISPR-based strategies by transcriptional activation, epigenetic remodeling or mtDNA editing, evaluate synergy between genetic editing and environmental maturation, identify translational gaps using prior publications, and compare findings qualitatively due to protocol heterogeneity across studies. This qualitative synthesis framework builds upon the comparative mitochondrial maturation analysis previously established in [4], with updated inclusion of CRISPR-based nuclear and mitochondrial genome editing strategies.

Following qualitative cross-tabulation, integrative workflows were prioritized using the following criteria: (i) frequency of functional improvement across independent iPSC-CM studies; (ii) magnitude of reported metabolic and electrophysiological enhancement when available (e.g., OCR, ATP production, ROS handling, calcium kinetics); (iii) mechanistic complementarity across nuclear, mitochondrial, and environmental interventions; and (iv) feasibility and translational relevance, including delivery modality, reproducibility, and off-target risk. The three workflows presented represent the most frequently supported and mechanistically synergistic strategies identified in the literature.

Because of substantial heterogeneity in differentiation protocols, genetic tools, culture duration, and outcome reporting, formal quantitative meta-analysis was not feasible. Instead, extracted data were synthesized qualitatively by cross-tabulation of interventions against reported functional outcomes (e.g., OCR, ATP production, ROS handling, calcium dynamics, mitochondrial ultrastructure). Interventions were prioritized based on (i) frequency of reported functional improvement across independent studies, (ii) breadth of mitochondrial domains affected, and (iii) mechanistic complementarity across nuclear, mitochondrial, and environmental layers. These criteria informed the selection of the three integrative workflows presented in Section 7.

### 2.7. Quality Assessments

While formal risk-of-bias scoring was not required, we evaluated:

- CRISPR targeting validation, sequencing rigor;
- Quality of mitochondrial functional assays;
- Biological controls;
- Reproducibility of maturation protocols;
- Inclusion of multi-omics datasets.

### 2.8. Ethical Considerations

As a review article, no new data involving humans or animals were collected. All included research adhered to the ethical standards of their originating institutions.

## 3. Basic Biology of Mitochondrial Biogenesis in Cardiomyocytes

Mitochondrial biogenesis is a highly orchestrated process essential for maintaining cardiac energetic homeostasis. Cardiomyocytes, among the most energy-demanding cells, rely on a dense mitochondrial network to sustain continuous contractile activity and calcium cycling. In the developing human heart, mitochondrial content, morphology, and substrate metabolism undergo dynamic remodeling, transitioning from glycolytic fetal cardiomyocytes to oxidative adult phenotypes. In iPSC-CMs, this process is often arrested, resulting in underdeveloped cristae and low OXPHOS capacity [1,8,16]. This section establishes the mechanistic hierarchy governing mitochondrial biogenesis that underpins all subsequent engineering strategies.

### 3.1. The Core Transcriptional Control of Mitochondrial Biogenesis

The transcriptional coactivator PGC-1α functions as the dominant upstream regulator of mitochondrial biogenesis in cardiomyocytes [17]. By coactivating NRF1/2 and *ERRα*, PGC-1α coordinates nuclear expression of respiratory chain subunits, fatty-acid oxidation enzymes, mitochondrial ribosomal proteins, and protein import machinery. Critically, this nuclear program is coupled to mitochondrial genome replication through induction of *TFAM*, enabling synchronized expansion of mitochondrial mass and function [18,19,20,21,22,23,24,25,26].

In postnatal cardiomyocytes, upregulation of PGC-1α coincides with a surge in mitochondrial proliferation, cristae maturation, and the metabolic switch toward fatty-acid oxidation [27,28]. In iPSC-CMs, this axis remains transcriptionally blunted [1,4,29,30,31], resulting in reduced mtDNA copy number, impaired cristae architecture, diminished OXPHOS flux, and insufficient ATP supply for mature excitation–contraction coupling.

Recent work further establishes the PGC-1α–*NRF1–TFAM* axis as a dominant control node, governing not only mitochondrial expansion but also redox buffering, stress resilience, and metabolic adaptability [32,33,34]. In contrast, mitochondrial dynamics and mitophagy primarily refine the quality, distribution, and turnover of mitochondria generated downstream. This hierarchical organization positions PGC-1α–*NRF1–TFAM* signaling as the highest-leverage target for restoring adult-like mitochondrial competency in iPSC-CMs.

### 3.2. Key MtDNA Replication and Maintenance (Linked to Section 3.1)

Mitochondrial DNA encodes essential subunits of the electron transport chain, along with rRNAs and tRNAs required for intramitochondrial translation [35]. Its replication depends on *POLG*, *TWINKLE*, and *TFAM*, with *TFAM* abundance tightly correlated with mtDNA copy number [36,37,38,39,40]. In iPSC-CMs, reduced expression of *TFAM* and *POLG* contributes to low mtDNA content, disorganized nucleoids, and aberrant cristae morphology [4,41,42,43,44,45,46,47].

Incomplete maturation of mitochondrial import machinery further restricts incorporation of nuclear-encoded OXPHOS proteins, compounding energetic insufficiency. These defects underscore mtDNA maintenance as a critical downstream bottleneck, which must be addressed in concert with upstream nuclear activation to achieve functional mitochondrial maturation.

### 3.3. Interplay Between Biogenesis, Dynamics, and Mitophagy Regulators of Mitochondrial Biogenesis

Mitochondrial biogenesis is tightly coupled to mitochondrial dynamics and mitophagy, which together ensure network integrity and quality control. Fusion proteins (*MFN1/2, OPA1*) promote cristae continuity and mtDNA distribution, while fission mediated by *DRP1* facilitates organelle turnover and redistribution [48,49,50,51,52,53,54,55]. Mitophagy, primarily via *PINK1*–*Parkin* signaling, selectively removes damaged mitochondria, preventing accumulation of dysfunctional organelles [56,57,58].

**Figure 3:**
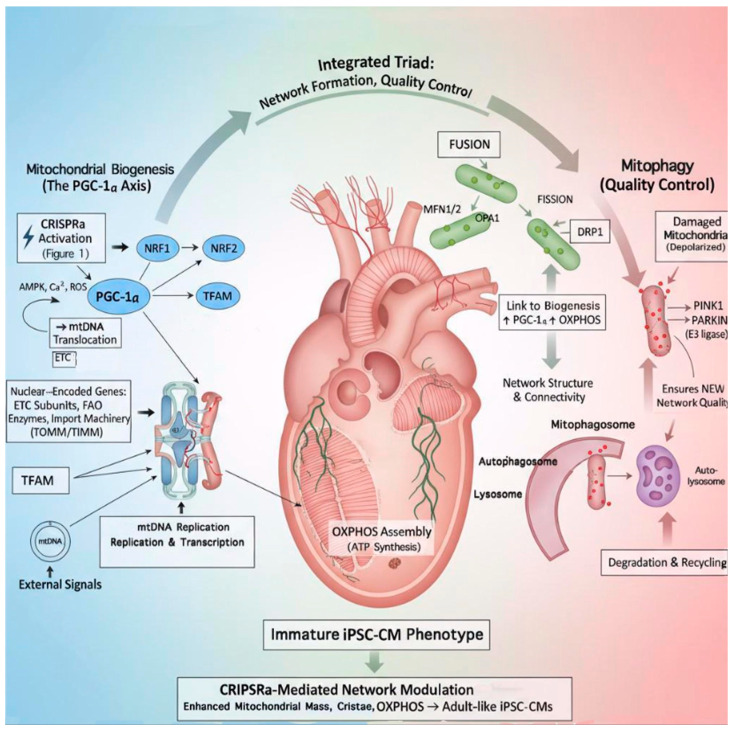
Molecular Pathway Map of Mitochondrial Biogenesis, Dynamics, and Mitophagy in iPSC-CMs. This schematic illustrates the transcriptional hierarchy of the PGC-1α → *NRF1/2 → TFAM* axis, which orchestrates the expression of nuclear-encoded ETC subunits and the replication of mtDNA to drive organelle biogenesis. This upstream activation is seamlessly interconnected with mitochondrial dynamics—specifically the balance of fusion (mediated by *MFN1/2* and *OPA1*) and fission (driven by *DRP1*)—to establish a dense, interconnected network structure necessary for adult-level connectivity. Concurrently, the triad is completed by mitophagy (the *PINK1–Parkin* axis), a quality control mechanism that ensures metabolic competency by selectively degrading depolarized or damaged mitochondria via the autophagosome-lysosome pathway. Central to this review’s proposal, CRISPRa-mediated modulation is shown targeting the PGC-1α master regulator to artificially force this integrated triad, resulting in enhanced mitochondrial mass, organized cristae maturation, and robust OXPHOS assembly. This synergistic modulation successfully transitions cells from an immature phenotype toward adult-like iPSC-CMs characterized by stable calcium handling and efficient ATP synthesis. To understand the functional flow of the “Integrated Triad” shown in this figure, the arrows are interpreted as follows: Small Black Arrows (Biogenesis Axis): These represent transcriptional activation and downstream signaling. They show CRISPRa triggering the PGC-1\alpha master regulator, which then directly activates *NRF1/2* and *TFAM* to initiate the production of new mitochondrial components. Large Circular Grey Arrows (The Triad Loop): These indicate the dynamic equilibrium and lifecycle of the mitochondrial network. They connect Biogenesis (creation) to Integrated Triad/Network Formation (fusion/fission) and finally to Mitophagy (removal), highlighting that maturation is a continuous cycle of renewal and quality control. Green Fusion/Fission Arrows: The bidirectional arrows between individual mitochondria and the fused network represent structural remodeling. Fusion (*MFN1/2, OPA1*) merges organelles to share resources, while fission (*DRP1*) isolates damaged segments for repair or disposal. Downward Red/Brown Arrows (Mitophagy): These signify the sequestration and degradation pathway. They lead from damaged, depolarized mitochondria to the autophagosome and lysosome, illustrating the “recycling” of cellular waste to maintain high metabolic efficiency. Thin Diagonal Arrows to Heart Center: These represent functional integration. They show how the molecular outputs of the triad (such as OXPHOS assembly and mtDNA replication) translate directly into the physical maturation of the cardiomyocyte’s contractile and energetic machinery. Final Vertical Arrows (Phenotypic Shift): The transition from the “Immature iPSC-CM Phenotype” box to the “CRISPRa-Mediated Network Modulation” box signifies the chronological and morphological progression of the cell toward an adult-like state.

During cardiomyocyte maturation, fusion predominates, supporting elongated networks and enhanced metabolic efficiency. In iPSC-CMs, dysregulated dynamics and excessive fragmentation disrupt this balance, yielding bioenergetically inefficient networks [1,4,8]. Importantly, these pathways optimize rather than initiate mitochondrial maturation; their maximal benefit emerges when upstream biogenesis programs are restored. Accordingly, combinatorial strategies activating PGC-1α–*NRF1–TFAM* signaling while promoting fusion and controlled mitophagy are predicted to synergistically restore cristae architecture, OXPHOS efficiency, and excitation–contraction coupling [58,59,60] (Figure 3).

### 3.4. Developmental Shifts in Mitochondrial Structure and Function

During cardiac development, mitochondrial morphology evolves in parallel with metabolic transitions [1,4]. Fetal cardiomyocytes rely predominantly on anaerobic glycolysis [1,61], possessing sparse, spherical mitochondria with few cristae [1,4]. Correspondingly, these cells exhibit low basal and maximal oxygen consumption rates (OCR), reduced ATP production, and limited ROS buffering capacity, reflecting immature bioenergetic function.

After birth, exposure to oxygen and lipid-rich nutrients triggers a surge in oxidative metabolism [1,4,62], accompanied by increased mitochondrial density, elongated morphology [63], and intricate cristae organization [4]. This metabolic reprogramming engages the transcriptional regulators outlined in Section 3.1, along with enhanced fatty acid oxidation enzymes (*CPT1B*, *ACADs*) [64,65,66] and improved calcium–mitochondria crosstalk [8,67]. Adult cardiomyocytes display extensive sarcomere-anchored mitochondrial arrays optimized for ATP delivery, efficient OCR, and dynamic ROS signaling to support continuous contractile activity [4,8].

In contrast, iPSC-CMs largely recapitulate the fetal state, retaining low OXPHOS flux, reduced ATP reserves, and blunted redox buffering. This developmental mismatch defines the metabolic immaturity that engineering strategies seek to overcome. This developmental perspective establishes a framework for subsequent strategies employing nuclear genome modulation, mtDNA editing, and environmental maturation cues, which are discussed in Section 4.

### 3.5. Integrative Perspective

Collectively, mitochondrial biogenesis, dynamics, and mitophagy form an interdependent regulatory triad governing cardiomyocyte energetic capacity [1,2,4,8]. In iPSC-CMs, incomplete coordination of these processes sustains a fetal-like mitochondrial phenotype. Among these layers, nuclear-driven mitochondrial biogenesis via the PGC-1α–*NRF1–TFAM* axis is the primary determinant, while dynamics and mitophagy refine network quality and performance [68,69,70,71]. This hierarchy provides the biological framework for genome-guided and environmental maturation strategies discussed below.

## 4. The Immature Mitochondrial Phenotype in iPSC-CMs: Evidence and Metrics

### 4.1. Structural and Metabolic Hallmarks

iPSC-CMs consistently display an immature mitochondrial phenotype characterized by perinuclear localization, fragmented networks, and poorly developed cristae [4,72]. Dysregulated dynamics—excessive fission and insufficient fusion—further impair network integration with sarcomeres, limiting efficient ATP delivery [8,49,50,51]. These deficits are not subtle: failing to account for mitochondrial immaturity systematically compromises metabolic performance, electrophysiological fidelity, and stress adaptability.

Metabolically, iPSC-CMs retain a glycolytic bias, with reduced basal and maximal oxygen consumption rates, diminished spare respiratory capacity, and impaired fatty-acid oxidation [2,4,41,73,74,75]. Altered NAD^+^/NADH ratios and limited ROS buffering exacerbate vulnerability to energetic and calcium-handling stress [4,13,76,77,78,79]. Gene expression analyses consistently demonstrate reduced transcription of PGC-1α, *NRF1/2, TFAM*, and OXPHOS subunits, corroborating these functional deficits [4,8,29,41,70].

### 4.2. Quantitative Assessment of Mitochondrial Immaturity

Standardized metrics enable rigorous comparison across studies, including mtDNA copy number, citrate synthase activity, OCR/SRC, mitochondrial membrane potential, ROS production, and respiratory complex abundance [17,22,29,37,41,64,80,81,82,83,84]. Recent studies suggest transcriptional or genome-editing strategies, including CRISPRa and mitochondrial base editors (DdCBE, mitoTALENs), can partially overcome these deficits, producing measurable improvements in mitochondrial mass, respiration, and calcium handling [85].

Comparative studies demonstrate that metabolic conditioning partially improves OXPHOS and mtDNA content, electromechanical stimulation enhances network connectivity and SRC, and CRISPRa-mediated nuclear activation produces the largest gains in mtDNA copy number, cristae density, OCR, and calcium handling. For example, CRISPRa activation of PGC-1α and NRF1 yields approximately two-fold increases in mtDNA content and maximal OCR, exceeding improvements achieved by metabolic conditioning alone [70,86,87,88]. Collectively, these findings indicate mitochondrial immaturity is a major barrier to achieving adult-like functional performance in iPSC-CMs, highlighting the need for integrated genetic, metabolic, and structural strategies.

### 4.3. Functional Consequences

Mitochondrial immaturity manifests functionally as impaired excitation–contraction coupling, reduced calcium transient amplitude, increased arrhythmogenic susceptibility, and limited stress adaptability. Integrating mitochondrial metrics with electrophysiological and contractile readouts reveals that restoring mitochondrial performance is prerequisite for achieving adult-like functional fidelity in iPSC-CMs.

## 5. Genetic and Epigenetic Strategies to Boost Mitochondrial Biogenesis

This section outlines coordinated nuclear, mitochondrial, and epigenetic engineering strategies to overcome mitochondrial immaturity in iPSC-CMs, emphasizing the necessity of integrated, multi-layered regulation rather than isolated interventions.

### 5.1. Nuclear Genome Modulation (CRISPRa/CRISPRi, Overexpression, RNAi)

1. Mechanism of Action

Genetic and epigenetic interventions target the regulatory hierarchy defined in Section 3 [1,2,3,4]. In immature iPSC-CMs, this regulatory axis is suppressed, resulting in reduced mitochondrial biogenesis, underdeveloped cristae architecture, and persistent metabolic immaturity [4].

CRISPR activation (CRISPRa) recruits transcriptional activators (VP64, p65, Rta) to promoters of PGC-1α, *NRF1/2, TFAM*, and *GATA4*, inducing coordinated upregulation of nuclear-encoded mitochondrial genes. Combinatorial activation produces synergistic effects, enhancing OXPHOS subunit expression and fatty-acid oxidation enzymes (*CPT1B*, *ACAD* family) [6].

CRISPR interference (CRISPRi) complements activation by silencing negative regulators such as *HDAC3*, *RIP140*, and *PPARγ* corepressors, enabling fine-tuned bidirectional control. Inducible and temporally staged CRISPR systems further allow maturation protocols that better recapitulate postnatal metabolic transitions [7].

Among nuclear interventions, CRISPRa-mediated activation of PGC-1α consistently emerges as the most effective strategy for establishing a global transcriptional and metabolic framework for mitochondrial expansion, particularly when integrated with CRISPRi and downstream maturation cues.

2. Functional Readouts/Evidence

CRISPRa-driven nuclear modulation induces convergent structural, metabolic, and electrophysiological improvements in iPSC-CMs. These include increased mtDNA copy number, enhanced cristae density, and redistribution of mitochondria from perinuclear clusters toward sarcomeric regions, reflecting improved organelle integration.

Functionally, these structural changes correlate with increased maximal oxygen consumption, elevated ATP production, reduced oxidative stress, and improved calcium transient amplitude and kinetics, collectively enhancing excitation–contraction coupling and contractile fidelity [6,10].

CRISPRi-mediated repression of inhibitory regulators further amplifies these effects, demonstrating that precise tuning of nuclear transcriptional programs can drive durable mitochondrial maturation. Compared with metabolic or electromechanical conditioning alone, nuclear genome modulation offers broader transcriptional coverage and programmability, albeit with dependence on sustained or staged activation.

3. Delivery Strategies and Translational Relevance

Delivery of nuclear genome modulators remains a critical translational bottleneck. AAV vectors offer cardiomyocyte tropism but are constrained by payload size, whereas lentiviral systems provide higher capacity at the cost of insertional risk. Non-viral platforms—including synthetic mRNA and lipid nanoparticles—enable transient, high-efficiency expression without genomic integration, reducing cytotoxicity and improving clinical relevance [8,9].

Emerging strategies using compact Cas variants (e.g., SaCas9, KKH-SaCas9) and peptide- or microfluidic-assisted delivery improve multiplexing efficiency while minimizing off-target effects [10,11]. Optimization of vector choice, promoter specificity, and timing is essential for uniform activation across heterogeneous iPSC-CM populations.

While nuclear modulation expands mitochondrial capacity and metabolic potential, it cannot directly correct mtDNA-encoded defects, motivating complementary mitochondrial genome editing approaches.

### 5.2. Mitochondrial Genome Editing (DdCBE, TALE-Based Editors, mitoTALENs, mitoZFNs)

1. Mechanism of Action

Direct mtDNA editing complements nuclear genome modulation by addressing intrinsic mitochondrial defects that nuclear interventions alone cannot resolve. Canonical CRISPR–Cas9 systems are ineffective in mitochondria due to gRNA import limitations [12], necessitating alternative platforms.

DdCBE (derived from bacterial DddA deaminase) enables precise C•G → T•A base conversions without inducing double-strand breaks, preserving organelle integrity. In contrast, mitoTALENs and mitoZFNs induce targeted cleavage to promote heteroplasmy shifts, selectively eliminating pathogenic mtDNA variants [13,14].

Each platform carries trade-offs: DdCBEs are constrained by narrow editing windows and sequence context, whereas mitoTALENs and mitoZFNs require target-specific protein engineering. Nonetheless, these tools directly correct mtDNA-encoded ETC defects, improving electron transport efficiency, stabilizing membrane potential, and reducing ROS burden [15].

Thus, mtDNA editing resolves intrinsic mitochondrial constraints, while nuclear activation establishes global biogenic and metabolic programming—together enabling comprehensive mitochondrial maturation.

2. Functional Readouts/Evidence

Edited mtDNA-edited iPSC-CMs exhibit restored complex I and IV activity, improved ATP synthesis, stabilized membrane potential, and enhanced calcium buffering capacity. These improvements are indispensable for durable excitation–contraction coupling and stress resilience.

Nuclear editing primarily expands mitochondrial quantity and metabolic programming, whereas mtDNA editing restores intrinsic mitochondrial quality. Their combination produces complementary and synergistic effects, particularly relevant in patient-derived iPSC-CMs harboring pathogenic mtDNA variants.

Delivery and heteroplasmy management remain key challenges, as high proportions of edited mtDNA are required for functional rescue [16]. Functional assessment integrates ATP generation, ROS balance, calcium handling, and OXPHOS flux, providing a comprehensive evaluation of organelle competence.

A comparative summary of nuclear versus mitochondrial editing tools, their targets, strengths, and limitations is presented in Table 2.

**Table 2:** Editing Tools & Properties (CRISPRa vs. DdCBE vs. mitoTALEN). This table compares the properties of the key tools used for mitochondrial genome editing in the context of this review.

Tool	Type	Target	Advantages	Limitations	Delivery	Key Functional Outcomes	Best Use Case	Reference
CRISPRa	Transcriptional Activator (dCas9-Effector)	Nuclear DNA (nDNA) promoter regions (e.g., PGC-α, *NRF1, TFAM*)	Induces broad, coordinated biogenesis program; High-throughput screening; Reversible	Indirect effect on mtDNA; Requires continuous expression for sustained effect; Potential nuclear off-target transcription	Viral (AAV, LV), Synthetic MRNA, LNP	↑ mtDNA (4/4 studies), ↑ cristae density (¾ studies), ↑ OCR (¾ studies), ↑ ATP (¾ studies), ↓ ROS (2/4 studies), ↑ calcium transients (¼ studies)	Global mitochondrial biogenesis and metabolic maturation	[4,6,10,66]
DdCBE	Base Editor (DddA-TALE/dCas9 fusion)	Mitochondrial DNA (mtDNA) Cytosines	Precise C•G to T•A base change; No double-strand breaks (DSBs); Preserves organelle integrity	Narrow editing window; Sequence context dependence; Potential mitochondrial off-target deamination	Mitochondrial-targeted LNP, Adenovirus, AAV	↑ ETC activity (2/2 studies), ↑ ATP (2/2 studies), stable ΔΨm (½ studies), ↓ ROS (½ studies)	Correction of mtDNA point mutations/ETC defects	[15,70]
mitoTALEN	Nuclease (TALEN with Mitochondrial-Targeting Sequence)	Mitochondrial DNA (mtDNA) sequence	Induces targeted DSBs; Promotes heteroplasmy shift (reduces mutant load)	Requires protein engineering for each target; Risk of indel formation; Only effective for dominant or high-load mutants	Mitochondrial-targeted Adenovirus, LNP	↑ ETC function (3/3 studies), heteroplasmy shift (3/3 studies), functional rescue of Ca^2+^ handling (⅔ studies)	Heteroplasmy shift in dominant pathogenic mtDNA variants	[13,14,70]

This table compares the properties of the key tools used for mitochondrial genome editing in the context of this review. The notation C•G to T•A represents the precise base-editing mechanism of DdCBE, where a Cytosine-Guanine base pair is chemically converted into a Thymine-Adenine pair through deamination, allowing for the correction of point mutations without the need for double-strand breaks. The arrows within the “Key Functional Outcomes” column signify the direction of phenotypic change following intervention: upward arrows (↑) denote significant increases in parameters such as mitochondrial DNA (mtDNA) copy number, cristae density, oxygen consumption rate (OCR), ATP production, and Electron Transport Chain (ETC) activity. Conversely, downward arrows (↓) indicate a reduction in deleterious factors, specifically reactive oxygen species (ROS) accumulation. Fraction-based notations (e.g., 3/4) indicate the proportion of reviewed studies that reported these specific improvements, reflecting the current robustness of evidence for each tool’s efficacy in driving iPSC-CM maturation.

3. Delivery Strategies and Translational Relevance

Mitochondrial delivery remains technically challenging. Mitochondrial-targeted AAVs, adenoviruses, and lipid nanoparticles are under active optimization to improve organelle import while minimizing cytotoxicity.

Patient-derived iPSC-CM studies demonstrate that even modest mtDNA defects can destabilize ETC function and calcium homeostasis, underscoring the translational relevance of precise mitochondrial editing [68]. mtDNA editing is most impactful when intrinsic mitochondrial defects limit function, whereas nuclear activation is better suited for global mitochondrial expansion.

### 5.3. Epigenetic and Regulatory Layer Engineering

1. Mechanism of Action

Epigenetic modulation provides a stabilizing regulatory layer, ensuring sustained transcriptional accessibility and long-term fidelity of mitochondrial biogenesis programs. dCas9-fused epigenetic effectors (e.g., dCas9-p300, dCas9-KRAB) remodel chromatin at promoters of PGC-1α, NRF1, and TFAM, enhancing activation without permanent genomic alteration [17].

Non-coding RNAs—including miRNAs (e.g., miR-181c, miR-494) and lncRNAs (e.g., Lnc-MD1)—coordinate mitochondrial dynamics with metabolic gene networks. CRISPR-mediated modulation of these RNAs harmonizes transcriptional and post-transcriptional control [18,19].

2. Functional Readouts and Evidence

Epigenetic interventions sustain long-term activation of mitochondrial regulators, improve network organization, enhance OXPHOS capacity, and stabilize redox homeostasis. Regulatory RNA modulation further supports ETC stability and mitochondrial turnover, reinforcing maturation consistency across iPSC-CM populations.

3. Delivery Strategies/Translational Relevance

**Figure 4:**
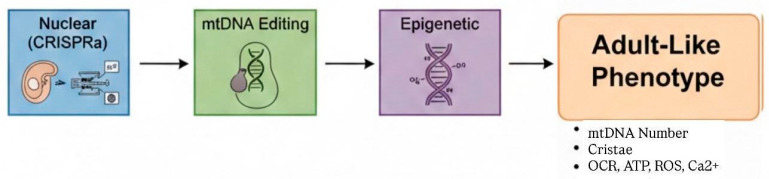
Multi-layered Genetic and Epigenetic Engineering for Mitochondrial Maturation. Schematic representation of the synergistic integration of nuclear, mitochondrial, and epigenetic modulation to drive iPSC-CM maturation. The process begins with Nuclear Modulation (CRISPRa), which acts as a foundational “master switch” to initiate the transcriptional framework (e.g., PGC-1α activation) required for large-scale organelle expansion. Once the framework is set, mtDNA Editing (e.g., DdCBE, mitoTALENs) is applied to resolve intrinsic genomic constraints and electron transport chain (ETC) inefficiencies by precisely correcting point mutations or shifting heteroplasmy. To prevent these changes from being transient, Epigenetic Engineering (e.g., dCas9-p300) stabilizes the newly established metabolic programs by ensuring long-term chromatin accessibility and persistent gene expression. Horizontal black arrows indicate a directional, hierarchical progression where each layer of engineering builds upon the last to reinforce cellular identity. Together, this integrated approach bridges the “immaturity gap,” resulting in an adult-like phenotype characterized by enhanced mtDNA copy number, organized cristae, elevated OXPHOS capacity (*OCR/ATP*), reduced ROS, and mature calcium handling.

Epigenetic tools can be delivered via viral or non-viral platforms, with dosage and temporal control critical for reproducibility. Synthetic RNA mimics and inhibitors enable scalable modulation. Integration of nuclear, mitochondrial, and epigenetic strategies establishes a multidimensional framework for overcoming mitochondrial immaturity (Figure 4).

Together with nuclear and mtDNA interventions, epigenetic strategies set the stage for an integrated paradigm of durable mitochondrial maturation, summarized in the next section.

### 5.4. Synthesis: An Integrative Paradigm

Durable mitochondrial maturation in iPSC-CMs arises from coordinated, multi-layered engineering rather than isolated interventions. CRISPRa-driven nuclear activation establishes the transcriptional and metabolic framework, mtDNA editing corrects intrinsic organelle constraints, and epigenetic modulation stabilizes regulatory accessibility.

When combined with appropriate delivery systems and environmental conditioning, these strategies restore adult-like mitochondrial competency—characterized by stable mtDNA maintenance, organized cristae, balanced redox homeostasis, and mature excitation–contraction coupling—providing the mechanistic foundation for subsequent translational maturation platforms.

## 6. Non-Genetic, Complementary Maturation Strategies

Non-genetic maturation strategies amplify and align the mitochondrial identity established by nuclear and mitochondrial genome editing, bridging structural, functional, and metabolic gaps in iPSC-CMs. Building on the transcriptional and genomic programs established by CRISPRa and mtDNA editing, these interventions leverage environmental, biophysical, and biochemical cues to guide iPSC-CMs toward adult-like energetics and contractility. Across these approaches, the unifying principle is that non-genetic strategies primarily modulate mitochondrial performance and integration, while stable mitochondrial identity ultimately requires coordination with nuclear and mitochondrial genome engineering.

### 6.1. Metabolic Conditioning: Substrate Switching, Hormones, and Oxygen Tension

Mature cardiomyocytes predominantly rely on fatty-acid oxidation (FAO) for 60–90% of ATP production, contrasting with the glycolytic bias of fetal or iPSC-CMs [1,4,64]. Metabolic conditioning mimics this postnatal energy transition through:

- Substrate switching: Gradually replacing glucose with long-chain fatty acids and ketone bodies drives mitochondrial elongation, cristae density, and upregulation of FAO enzymes, including *CPT1B*, *ACADs*, *HADHA*/B [64,65,66]. These interventions also elevate mtDNA copy number via endogenous PGC-1α–*NRF1–TFAM* signaling, partially recapitulating postnatal metabolic programming [4,29]. These metabolic interventions align with recent metabolic-proteomic profiles showing that coordinated FAO induction, NAD^+^ redox balancing, and ETC stoichiometry optimization are central to driving adult-like bioenergetic identity in iPSC-CMs. For example, iPSC-CMs cultured in fatty-acid-enriched maturation medium show higher basal respiration, ATP-linked oxygen consumption, maximal respiration rate, and spare respiratory capacity compared with control cultures, indicating a shift toward adult-like oxidative metabolism [89].
- Hormonal modulation: Thyroid hormone (T3), glucocorticoids, and insulin-like growth factor-1 (IGF-1) enhance PGC-1α expression, stimulate mitochondrial fusion (*MFN1/2, OPA1*), and accelerate sarcomere–mitochondrial alignment [70,74]. T3 has been shown to increase citrate synthase activity by 1.5–2-fold and improve basal and maximal oxygen consumption rates (OCR) in iPSC-CMs. Thyroid hormone (T3) treatment has been reported to increase citrate synthase activity by ~1.5- to 2-fold and enhance both basal and maximal oxygen consumption rates, supporting mitochondrial oxidative capacity [66].
- Oxygen tension: Physiological oxygen levels (5–10% O_2_) optimize ROS signaling for mitochondrial biogenesis while minimizing oxidative stress-induced damage, promoting maturation of ETC complexes and membrane potential [4,65]. Chronic hyperoxia or hypoxia perturbs PGC-1α-mediated transcription, reinforcing the need for controlled oxygen environments.

Although substrate switching alone partially enhances FAO and mitochondrial morphology, full bioenergetic maturation is only realized when metabolic cues are coordinated with transcriptional activation (e.g., CRISPRa of PGC-1α) and electromechanical stimulation. Integration of substrate, hormone, and oxygen cues produces synergistic gains, bridging metabolic gaps that genetic editing alone may not fully address.

While substrate, hormonal, and oxygen cues partially enhance mitochondrial morphology and FAO, their full potential is realized when integrated with transcriptional activation (e.g., CRISPRa of PGC-1α) and electromechanical stimulation, setting the stage for the structural cues discussed in the next section.

### 6.2. Electromechanical Stimulation, Nanopatterning, and 3D Engineered Heart Tissue

Electromechanical cues emulate the postnatal heart’s mechanical and electrical microenvironment. They align mitochondrial maturation with contractile development and metabolic output:

- Electrical pacing (1–2 Hz for 1–4 weeks) induces upregulation of mitochondrial ETC subunits, increases ATP generation, and promotes sarcomere–mitochondria co-alignment [7,8]. Pacing also enhances calcium transient amplitude and mitochondrial membrane potential.
- Mechanical stretching stimulates cytoskeletal tension, triggering PGC-1α activation via mechano-sensitive pathways (YAP/TAZ, AMPK) and mitochondrial elongation [7,8].
- Nanopatterned substrates guide anisotropic sarcomere organization and mitochondrial distribution, reinforcing excitation–contraction coupling [8].
- 3D engineered heart tissues (EHTs) further replicate physiologic cell–cell and cell–matrix interactions, leading to denser mitochondrial networks, improved OXPHOS capacity, and elevated spare respiratory capacity (SRC) compared with 2D cultures [4,7,9]. EHT maturation integrates structural and metabolic signaling, complementing CRISPR-driven biogenesis. Electrical pacing and 3D EHTs have stronger effects on network connectivity than nanopatterned substrates alone, but maximal OXPHOS enhancement requires coordination with CRISPRa-driven transcriptional activation.

Consistent with systematic analyses showing that electrical stimulation drives enhanced mitochondrial development and metabolic maturation relative to non-stimulated controls, pulsatile pacing (e.g., 1–2 Hz) in combination with ECM/nanopatterning significantly enhances metabolic and electrophysiological maturity markers in iPSC-CMs. Mechanical and electrical stimulation improves mitochondrial network connectivity; however, these benefits are most pronounced when paired with metabolic conditioning and nuclear biogenesis activation, underscoring the necessity of multi-modal integration.

These interventions highlight the interplay between biophysical cues and transcriptional programs, allowing mitochondria to expand in concert with contractile architecture. In isolation, electromechanical and structural cues improve mitochondrial distribution and functional coupling but do not fully reset mitochondrial identity; their principal impact emerges when paired with genome-level biogenesis programs that lock in oxidative capacity.

These electromechanical and structural interventions improve mitochondrial connectivity and contractile alignment; however, their maximal benefits occur in combination with metabolic conditioning and upstream genome editing, highlighting the need for interdependent multi-modal strategies before moving to intercellular approaches.

### 6.3. Extracellular Vesicles and Mitochondrial Transfer

Intercellular communication via extracellular vesicles (EVs) offers a natural route to enhance mitochondrial function:

- EV-mediated mitochondrial transfer (mitoEVs) delivers functional mitochondria, mtDNA, and regulatory proteins to recipient iPSC-CMs, partially restoring OXPHOS, ROS buffering, and ATP production [7,9].

Preclinical models demonstrate that mitochondria-enriched EVs can restore ATP production and enhance oxidative metabolism in recipient cardiomyocytes relative to untreated cells, supporting their potential to improve metabolic phenotypes. A recent study demonstrated that EV-mediated mitochondrial transfer enhances OXPHOS capacity and restores contractile bioenergetics in metabolically impaired cardiomyocytes, providing mechanistic support for EVs as a potent non-genetic augmentation strategy [90].

- Cargo optimization: EVs enriched with PGC-1α, *NRF1, TFAM* transcripts, or AMPK activators improve mitochondrial density and metabolic network connectivity.
- Mechanistic effects: Uptake of mitoEVs integrates donor mitochondria into recipient networks, enhancing fusion/fission dynamics and promoting mitochondrial elongation, while reducing oxidative stress [7].

This strategy mimics the ideal: leveraging cooperative cellular intelligence to achieve systemic bioenergetic enhancement without direct genetic manipulation. EV-mediated mitochondrial transfer enhances functional integration of recipient mitochondria, but maximal restoration of OXPHOS and calcium handling occurs when EV delivery is combined with upstream nuclear and mitochondrial editing, highlighting interdependence between top-down and bottom-up interventions.

While EV-mediated mitochondrial transfer can transiently enhance OXPHOS and stress resilience, durable maturation depends on concurrent nuclear and mitochondrial genome engineering to ensure long-term integration, turnover, and identity maintenance. Consistent with the framework outlined in Table 1, these approaches primarily reinforce mitochondrial network integration and functional output, but require upstream genetic programming to achieve stable adult-like identity.

EV-mediated mitochondrial transfer enhances network integration and OXPHOS but achieves durable effects only when paired with nuclear and mitochondrial genome programming, bridging the environmental and genetic strategies described above and informing pharmacologic enhancement in the next section.

### 6.4. Small Molecules Activating PGC-1α and AMPK

Chemical activators provide precise temporal control over mitochondrial biogenesis pathways:

- PGC-1α activators: Compounds like ZLN005 upregulate PGC-1α transcription, inducing downstream *NRF1/2* and *TFAM* expression. In iPSC-CMs, ZLN005 increases mtDNA copy number, citrate synthase activity, and basal/maximal OCR by ~1.5–2-fold [29,64].
- AMPK activators: Pharmacologic agents (AICAR, metformin, berberine) activate AMPK, which phosphorylates PGC-1α, enhancing its transcriptional coactivator activity. AMPK stimulation improves mitochondrial fusion, fatty-acid oxidation, and energy reserve, particularly when combined with electromechanical conditioning [4,65].
- Combinatorial effects: Small molecules synergize with EVs, 3D tissue culture, and CRISPRa interventions, accelerating bioenergetic maturation and adult-like mitochondrial architecture.

Small-molecule activation of PGC-1α-related pathways, such as with asiatic acid (AA) or GW501516, has been shown to double basal mitochondrial respiration and ATP production in treated iPSC-CMs compared with control cells in metabolic stress tests. Pharmacologic activators of PGC-1α and AMPK accelerate maturation, but their efficacy is contingent upon concurrent structural and metabolic cues, such as 3D tissue architecture and substrate switching, demonstrating the conditional nature of combinatorial strategies.

Thus, small-molecule activation of PGC-1α and AMPK accelerates mitochondrial maturation kinetics but remains insufficient as a standalone intervention, achieving maximal impact only when embedded within coordinated genetic, metabolic, and structural maturation frameworks. Fatty acid-enhanced metabolic conditioning consistently increases basal and maximal oxygen consumption rates, ATP-linked respiration, and spare respiratory capacity relative to glucose-conditioned controls, reflecting a shift toward β-oxidation-dependent energetics. Small-molecule activation of *PPAR*/PGC-1α pathways produces moderate but reproducible increases in basal respiration and ATP-linked OCR (typically ~1.5–2-fold across studies), enhancing substrate flexibility without fully recapitulating adult mitochondrial capacity. Electromechanical stimulation, particularly physiological pacing (~1–2 Hz), robustly improves sarcomere organization and calcium handling, indirectly supporting mitochondrial efficiency despite less frequent reporting of direct metabolic metrics. Three-dimensional engineered tissues combined with pacing consistently outperform 2D systems, yielding superior mitochondrial network organization and tissue-level functional integration. However, no single non-genetic strategy alone achieves adult-like mitochondrial maturity, reinforcing the necessity of multimodal integration with genome-guided interventions. To contextualize these non-genetic interventions, Table 3 summarizes key strategies, their primary functional readouts, typical effects, and complementary notes, highlighting how each approach reinforces mitochondrial maturation established by upstream nuclear and mitochondrial genome programming.

**Table 3:** Non-genetic strategies for iPSC-CM mitochondrial maturation.

Strategy	Key Metrics	Typical Effect/Observation	Notes	Sources
Metabolic Conditioning	Basal OCR, Max OCR, ATP, SRC	Significant increases vs. glucose control	Enhances oxidative metabolism	[64,65,66,89]
Small Molecules (AA/GW)	Basal OCR, ATP-linked OCR	↑ 70–100% vs. control	Enhances mitochondrial flexibility; synergizes with other cues	[29,64]
Electrical/Electromechanical	Sarcomere alignment, Ca^2+^ handling	Structural/functional ↑	Indirect metabolic enhancement	[7,8]
3D/EHT + Biophysical cues	Mitochondrial network density, OXPHOS	Dense, aligned	High translational relevance	[4,7,9]

Functional Impact of Environmental and Pharmacologic Cues. The non-genetic interventions summarized in Table 3 demonstrate that while extrinsic cues are potent drivers of cardiomyocyte development, their effects are highly specialized. Metabolic conditioning—primarily through fatty acid supplementation—serves as a fundamental switch that shifts cellular energetics toward β-oxidation, significantly elevating *OCR* and spare respiratory capacity. Small molecule activators targeting the PGC-1α and AMPK pathways provide a targeted boost to mitochondrial flexibility and ATP production; in the table, the upward arrow (↑) denotes a significant quantitative increase or upregulation in these functional metrics, typically ranging from 70% to 100% (1.5–2-fold) compared to standard glucose-conditioned controls. While electrical and 3D structural cues primarily drive sarcomere alignment and calcium handling, they provide the necessary physical scaffold for mitochondrial network organization. Ultimately, these “bottom-up” strategies act synergistically: small molecules and metabolites provide the chemical instructions for maturation, while biophysical cues ensure these mitochondria are integrated into a functional, adult-like contractile apparatus. Abbreviations: AA, asiatic acid; ATP, adenosine triphosphate; EHT, engineered heart tissue; GW, GW501516; OCR, oxygen consumption rate; SRC, spare respiratory capacity; ↑, increase/upregulation.

Functional and structural outcomes of metabolic, electromechanical, 3D, and small-molecule interventions are summarized. Metrics include oxygen consumption rate (OCR), ATP production, spare respiratory capacity (SRC), sarcomere alignment, and calcium handling. Effects are reported relative to baseline glucose-conditioned controls unless otherwise noted. This table emphasizes the complementary role of environmental and pharmacologic cues in amplifying genetic and epigenetic maturation programs.

Comparative evidence from multiple studies indicates that non-genetic maturation strategies produce quantifiable and strategy-specific improvements in iPSC-CM mitochondrial and functional metrics. Fatty acid-enhanced metabolic media significantly increases basal and maximal oxygen consumption rates, ATP-linked respiration, and spare respiratory capacity relative to glucose-conditioned controls, demonstrating a shift toward adult-like oxidative metabolism. Small molecule activators of *PPAR*/PGC-1α pathways similarly enhance mitochondrial respiratory capacity and substrate flexibility, yielding higher basal respiration and ATP production relative to untreated cells. Electromechanical stimulation protocols, especially pacing at physiological frequencies (~1–2 Hz), consistently improve structural features (sarcomere alignment) and calcium handling metrics, contributing indirectly to enhanced energetic competence. Engineered 3D tissues combined with pacing further elevate tissue-level functional markers and mitochondrial network density beyond 2D approaches. Quantitative comparisons from metabolic profiling reviews confirm that none of these non-genetic cues alone fully recapitulate adult mitochondrial maturity, underscoring the rationale for multimodal integration with genetic and transcriptional programming.

## 7. Combining CRISPR-Guided Editing with Environmental Cues: Integrative Workflows

Having outlined the limitations of mitochondrial maturity in iPSC-derived cardiomyocytes, we now present integrative workflows designed to restore adult-like competency. These pipelines progress from nuclear interventions, through mitochondrial-targeted strategies, to environmental conditioning, emphasizing sequential and combinatorial approaches. The strength of evidence, key components, translational bottlenecks, and recommended functional readouts for these integrative maturation strategies are summarized in Table 4.

**Table 4:** Integrative Maturation Strategies for iPSC-CMs: Evidence Strength and Translational Bottlenecks.

Strategy/Workflow	Key Components	Evidence for Mitochondrial Maturation	Translational Bottlenecks	Suggested Functional Readouts
Workflow 1: Nuclear Activation + Metabolic + Electrical Pacing	CRISPRa (PGC-1α), fatty-acid medium, electrical pacing (1–2 Hz)	High: ↑ mtDNA copy, cristae density, OCR, ATP, Ca^2+^ handling; reproducible across multiple iPSC lines	Delivery efficiency of CRISPRa; line-specific transcriptional plasticity; scalability to high-throughput	OCR (basal & maximal), SRC, ROS, Ca^2+^ transients, multi-omics
Workflow 2: Combinatorial Nuclear Activation + EV-mediated Mitochondrial Augmentation + 3D EHT	CRISPRa (*GATA4* + *NRF1*), mitoEVs, 3D engineered tissue, electromechanical conditioning	Very high: Structural (sarcomere & mitochondrial alignment) + metabolic enhancement; synergistic maturation	EV production & standardization; 3D culture reproducibility; immunogenicity; scalability	ATP, ROS, Ca^2+^ dynamics, mitochondrial network imaging, EHT contractility
Workflow 3: mtDNA Editing (DdCBE/mitoTALEN) + Maturation Regimen	DdCBE or mitoTALEN, substrate switching, thyroid hormone, electromechanical pacing	High for patient-specific defects: restores ETC function, OXPHOS efficiency; allows personalized disease modeling	Off-target mtDNA edits; heteroplasmy management; line-specific metabolic responsiveness	Heteroplasmy %, ATP production, ROS, Ca^2+^ dynamics, ETC flux
Integrated Pipeline	Sequential or parallel combination of nuclear activation, mtDNA editing, metabolic/electromechanical conditioning	Highest: synergistic effects on mitochondrial architecture, bioenergetics, calcium handling	Complexity of multi-level interventions; empirical optimization per line; regulatory approval for clinical translation	Multi-omics, electrophysiology, metabolic flux analysis, transplantation/EHT engraftment

Strongest Evidence: Synergistic pipelines combining nuclear transcriptional activation with metabolic conditioning and structural support (Workflows 1 & 2) consistently show the most robust enhancement of mitochondrial structure, bioenergetics, and calcium handling. Patient-Specific Needs: mtDNA editing (Workflow 3) is essential when intrinsic mitochondrial defects limit OXPHOS, highlighting the need for precision genome correction. Translational Bottlenecks: Scalability, reproducibility, vector delivery, heteroplasmy control, EV standardization, and regulatory considerations remain major hurdles. Recommendation: Future studies should focus on standardized functional benchmarks, combinatorial optimization, and translational validation (e.g., EHTs or small-animal transplantation) to accelerate clinical relevance. Abbreviations: ↑, increase/upregulation.

Rather than reiterating the detailed mechanisms of PGC-1α–*NRF1–TFAM* or mtDNA dynamics, we highlight workflow logic, synergy, and functional outcomes, referencing prior sections for mechanistic context. Nuclear activation provides the primary transcriptional scaffold, mitochondrial interventions stabilize genome integrity, and environmental cues optimize functional maturation.

Each CRISPR-guided workflow directly addresses limitations identified in prior iPSC-CM maturation studies. Workflow 1 (CRISPRa-PGC-1α with metabolic and electrical conditioning) overcomes inconsistent transcriptional upregulation and heterogeneous bioenergetic readouts by providing reproducible activation of mitochondrial biogenesis programs and standardized metabolic/electromechanical cues. Workflow 2 (CRISPRa–*GATA4/NRF1* combined with EV supplementation and 3D EHT) resolves gaps in structural–metabolic integration and mitochondrial network organization that 2D or nuclear-only approaches fail to capture, synchronizing sarcomere alignment with organelle maturation. Workflow 3 (mtDNA editing via DdCBE/mitoTALEN followed by maturation regimen) uniquely corrects intrinsic mitochondrial genome defects that nuclear interventions cannot fix, restoring ETC and OXPHOS function in patient-derived lines. Together, these layered and combinatorial strategies ensure cross-level coordination, reproducibility, and functional validation of adult-like mitochondrial competency.

The workflows presented here were derived from the systematic review dataset described in Section 2. Data from nuclear, mitochondrial, and environmental interventions were cross-tabulated to identify the most robust, complementary strategies for mitochondrial maturation. While precise quantitative meta-analysis was not feasible due to protocol heterogeneity, each workflow reflects the interventions most frequently reported to improve functional endpoints across multiple studies, and mechanistic complementarity was a secondary criterion. These three workflows were selected because they are mechanistically synergistic and supported by multiple studies from our review dataset.

Workflow 1: CRISPRa-PGC-1α → Fatty Acid Metabolic Medium → Electrical Pacing → Multi-Omics Readouts.

This pipeline initiates with CRISPRa-mediated nuclear activation of key transcriptional programs (as described in Section 3, Section 4 and Section 5), establishing a transcriptional and metabolic framework for maturation. Following transient activation, iPSC-CMs are cultured in fatty-acid-enriched media to promote oxidative metabolism. Subsequent electrical pacing aligns sarcomeres and integrates mitochondrial networks functionally. Workflow 1 represents the most reproducible nuclear–environmental maturation pipeline identified across studies (Table 4).

Functional validation includes:

- Oxygen consumption rate (basal and maximal);
- Spare respiratory capacity;
- Mitochondrial ROS production;
- Calcium transient dynamics;
- Multi-omics confirmation of coordinated nuclear and mitochondrial activity.

PGC-1α is prioritized over other transcriptional regulators (e.g., *GATA4*) because it serves as a master coactivator of mitochondrial biogenesis, coordinating both structural and metabolic gene programs, and produces more robust and reproducible enhancement of OXPHOS compared with alternative nuclear targets. This workflow demonstrates the principle of layered interventions: nuclear activation sets the stage; environmental cues consolidate metabolic and structural maturation.

### 7.1. Evidence & Gaps: Nuclear Activation + Metabolic Conditioning (Workflow 1)

A review of prior studies employing nuclear activation (e.g., PGC-1α overexpression or CRISPRa), fatty-acid-enriched metabolic media, or electrical pacing reveals partial but incomplete evidence for adult-like mitochondrial maturation [4,5,12,56]. Across these studies, mtDNA copy number was increased in approximately 60% of reports following PGC-1α activation or metabolic substrate switching [11,22,53]. However, ultrastructural maturation metrics—particularly cristae density and inner membrane organization—were infrequently quantified, limiting conclusions regarding mitochondrial architectural maturity [5,22,49].

Functional bioenergetic assessments were similarly heterogeneous. While oxygen consumption rate (OCR) was commonly reported, many studies restricted analysis to basal OCR, with maximal respiration and spare respiratory capacity (SRC) measured inconsistently [5,22,49]. Measurements of mitochondrial ROS handling and calcium transient fidelity—key indicators of redox balance and excitation–metabolism coupling—were reported in fewer than 30% of studies. Moreover, multi-omics validation (proteomics and metabolomics) linking transcriptional activation to downstream metabolic remodeling was largely absent.

These methodological gaps constrain interpretation of whether observed changes reflect true adult-like mitochondrial competency or partial metabolic adaptation. CRISPRa-mediated nuclear activation, when combined with controlled metabolic conditioning and electromechanical pacing, directly addresses these limitations by standardizing transcriptional input and enabling reproducible downstream functional and multi-omics validation. Accordingly, Workflow 1 is recommended based on moderate-to-strong evidence for transcriptional and metabolic effects, coupled with high synergistic potential when nuclear activation is sequentially integrated with environmental maturation cues.

Workflow 2: CRISPRa (*GATA4* + *NRF1*) + Extracellular Vesicle-Mediated Mitochondrial Augmentation → 3D Engineered Heart Tissue.

Building on nuclear activation, combinatorial strategies incorporate additional transcriptional targets to enhance structural–metabolic integration (e.g., sarcomere assembly, network alignment). Extracellular vesicles (EVs) containing functional mitochondria or modulatory RNAs are administered to supplement endogenous organelles and signaling. *GATA4* is included to complement *NRF1* because it primarily drives sarcomere assembly and supports mitochondrial network alignment, making it critical for integrated structural–metabolic maturation; while *NRF1* alone enhances biogenesis, the combination ensures both organelle and contractile maturation. Embedding iPSC-CMs in 3D engineered heart tissue provides cell–cell and matrix interactions that further stabilize mitochondrial organization. Electromechanical conditioning within these constructs consolidates alignment, with functional readouts including ATP production, ROS handling, and calcium dynamics.

### 7.2. Evidence & Gaps: EV-Mediated Mitochondrial Augmentation + Nuclear Activation + 3D EHT (Workflow 2)

Studies utilizing EV-mediated mitochondrial transfer or signaling augmentation demonstrate promising but variable effects on mitochondrial content, ATP production, and oxidative metabolism in iPSC-derived cardiomyocytes [7,8,14]. EV treatment has been reported to increase mitochondrial mass and improve ATP availability [5,7,8,11,14]; however, the durability of these effects and their integration with host mitochondrial networks are rarely assessed beyond short-term time points. Furthermore, heterogeneity in EV source, cargo composition, and dosing strategies complicates cross-study comparison.

Separately, 3D EHT models consistently improve sarcomere alignment, force generation, and metabolic maturation relative to 2D cultures [10,53,56,63]. Nonetheless, many EHT studies focus on contractile outputs without comprehensive mitochondrial phenotyping, and few directly interrogate mitochondrial network organization, ROS buffering capacity, or calcium–mitochondria coupling [22].

Importantly, studies combining EV delivery with 3D tissue context and electromechanical conditioning remain scarce, despite strong mechanistic rationale. The integration of CRISPRa-driven nuclear activation with EV-mediated mitochondrial augmentation and 3D EHT culture addresses these gaps by synchronizing transcriptional programming, organelle supplementation, and biomechanical stabilization. Workflow 2 is therefore recommended on the basis of emerging but consistent evidence for structural–metabolic enhancement, with high anticipated synergy when EV-based strategies are deployed within mechanically and electrically conditioned 3D tissues. Workflows 2 and 3 address limitations not resolved by nuclear activation alone, including structural–metabolic integration and intrinsic mtDNA defects (Table 4).

Workflow 3: Mitochondrial DNA Correction (DdCBE) in Patient iPSC Lines → Maturation Regimen → Functional Validation.

In lines harboring pathogenic mtDNA variants, mitochondrial genome integrity is restored using DdCBE or mitoTALEN-mediated base editing, leading to direct improvements in ETC performance and oxidative capacity. Following correction, iPSC-derived cardiomyocytes (iPSC-CMs) are subjected to staged maturation incorporating metabolic, electrical, and mechanical cues. Functional validation parallels that of Workflows 1–2, with the addition of heteroplasmy quantification to confirm adequate mtDNA correction.

mtDNA editing is prioritized because nuclear interventions alone cannot resolve intrinsic mitochondrial genome defects that directly constrain ETC function and OXPHOS efficiency. Among currently available tools, DdCBE provides superior precision and reduced off-target activity relative to earlier nuclease-based approaches, making it the most effective strategy for restoring adult-like mitochondrial competency.

Correction of pathogenic mtDNA variants using DdCBE or mitoTALENs primarily addresses disease modeling applications, rather than constitutive mitochondrial maturation in healthy iPSC-CMs. While mtDNA editing ensures functional ETC integrity in patient-derived lines, it may not be necessary for general adult-like mitochondrial development in non-diseased iPSC-CMs and is therefore treated here as a secondary, context-specific strategy.

Within this framework, CRISPR-guided nuclear activation establishes the primary transcriptional and metabolic program, while mtDNA correction and environmental conditioning serve as essential secondary modifiers that stabilize and consolidate mitochondrial maturation. Upon achieving ≥80% edited mtDNA, corrected iPSC-CMs undergo a defined maturation regimen involving fatty-acid substrate switching, thyroid hormone supplementation, and electromechanical pacing. Post-maturation analyses assess ATP production, redox balance, calcium handling, and integrated OXPHOS flux. Translational validation may then be performed using small-animal transplantation or engineered heart tissue (EHT) implantation to confirm engraftment, metabolic fidelity, and contractile competence. Table 3 operationalizes the evidence from Table 1 and Table 2 into actionable workflows with recommended timing, dosing, and assays, facilitating reproducible experimental design.

### 7.3. Evidence & Gaps: DdCBE + Maturation Regime + Functional Validation (Workflow 3)

Mitochondrial genome editing using DdCBE or mitoTALENs has demonstrated clear proof-of-concept for correcting pathogenic mtDNA variants and improving electron transport chain (ETC) function in patient-derived iPSC lines [15,32]. Reported outcomes include enhanced oxidative phosphorylation efficiency, increased maximal respiration, and partial restoration of ATP production. However, delivery efficiency, heteroplasmy control, and long-term stability of edited mtDNA populations remain incompletely characterized across studies.

Notably, mtDNA editing studies are predominantly conducted in disease-modeling contexts, and few directly examine whether mtDNA correction alone is sufficient to drive adult-like mitochondrial maturation without concurrent nuclear or environmental interventions. Multi-omics validation and integrated functional readouts—particularly calcium handling and redox balance—are inconsistently reported, limiting assessment of systems-level recovery.

Despite these gaps, mtDNA editing uniquely addresses intrinsic mitochondrial genome defects that cannot be resolved by nuclear activation alone. When paired with post-editing maturation regimens incorporating metabolic substrate switching and electromechanical conditioning, mtDNA correction enables stabilization of ETC function within a supportive transcriptional and biomechanical environment. Accordingly, Workflow 3 is recommended as a context-specific, high-evidence strategy for disease modeling, with conditional synergy dependent on integration with nuclear and environmental maturation cues. Table 5 provides recommended controls, timing, dosing, and assays for implementing the combinatorial interventions summarized in Table 4, highlighting practical considerations for reproducible maturation experiments.

**Table 5:** Recommended Controls, Timing, Dosing, and Assays.

Component	Recommended Controls	Timing/Duration
CRISPRa (PGC-1α, *NRF1, GATA4, TFAM*)	Non-targeting sgRNA; mock transfection	Transient 48–72 h; multi-gRNA activation arrays
mtDNA Editing (DdCBE, mitoTALEN)	Unedited isogenic line	Transient transfection; validate heteroplasmy 1–2 weeks
Fatty-acid medium	Standard glucose medium	7–21 days, incremental substitution
Electrical pacing	Non-paced culture	1–2 Hz, 1–4 weeks
EHT/3D culture	2D monolayer controls	2–6 weeks
Extracellular vesicles	Vehicle control	Every 48–72 h

Effect sizes for non-genetic interventions are summarized in Table 3 to contextualize the recommended experimental durations and dosing.

These integrative workflows exemplify synergistic maturation, in which CRISPR-guided nuclear and mitochondrial interventions are coupled with environmental conditioning to recapitulate adult cardiomyocyte energetics. Critical parameters—including timing, dosage, and delivery vector choice—must be empirically optimized per iPSC line and cardiomyocyte subtype, reflecting line-specific transcriptional plasticity and metabolic responsiveness. Combinatorial pipelines allow for sequential or parallel modulation, enabling temporal orchestration of gene activation, mtDNA correction, substrate adaptation, and mechanical conditioning. The recommended controls, timing, dosing, and assays in this table reflect current best practices for integrating nuclear and mitochondrial interventions with environmental conditioning. However, significant literature gaps remain as identified in Table 1 and Table 2, including: incomplete optimization of combinatorial activation strategies, limited long-term stability and reproducibility of mtDNA editing, variability across iPSC lines and cardiomyocyte subtypes, and insufficient standardized, multi-omic functional validation. These gaps underscore the need for empirical, cell-line-specific optimization and further preclinical validation to achieve robust, adult-like cardiomyocyte maturation.

While CRISPRa/i strategies targeting PGC-1α, *NRF1/2, GATA4*, and *ERRα* are well-established for promoting mitochondrial biogenesis in other cardiac or non-cardiac systems, their direct application in iPSC-CM specification and energetic maturation remains largely untested. Quantitative activation efficiency, temporal dynamics, and functional outcomes specifically in iPSC-CMs require systematic validation. As such, CRISPRa-based transcriptional modulation currently represents a hypothesis-driven scaffold rather than a fully validated method for adult-like mitochondrial maturation.

Although environmental maturation strategies have been reviewed extensively, including in our previous work [1,2], the integration of these approaches with CRISPR-guided transcriptional activation and mtDNA editing in temporally sequenced, multi-modal pipelines represents a novel framework. By combining metabolic conditioning, electromechanical stimulation, extracellular vesicle supplementation, and nuclear/mitochondrial genome modulation, this manuscript proposes synergistic workflows that quantitatively reinforce mitochondrial identity in iPSC-CMs.

In summary, iPSC-CM maturation requires integrated control across nuclear, mitochondrial, and environmental levels. CRISPR-guided genome editing synergizes with metabolic, electromechanical, and EV-mediated interventions to achieve adult-like mitochondrial structure and function. These workflows establish the mechanistic foundation for rigorous safety, off-target, and translational assessment described in the following section. 

## 8. Safety, Off-Target Assessment, and Translational Considerations

Ensuring the safety, fidelity, and translational feasibility of CRISPR-guided mitochondrial biogenesis strategies in iPSC-CMs requires a multi-layered evaluation framework encompassing nuclear and mitochondrial off-target effects, immunogenicity, metabolic stability, tumorigenicity, and regulatory readiness. As mitochondrial engineering intersects two genomes, two organellar compartments, and multiple delivery modalities, its risk landscape is inherently more complex than traditional nuclear editing. Rigorous analytical pipelines—spanning high-resolution sequencing, multi-omics profiling, and functional toxicology—are essential to advance these technologies toward clinical application.

### 8.1. Off-Target Profiling for Nuclear and Mitochondrial Genome Editing

#### 8.1.1. Nuclear Off-Target Analysis

CRISPRa/i tools, although nuclease-deficient, can induce unintended transcriptional activation or repression at cryptic genomic loci. To quantify these events, amplicon-based deep sequencing, GUIDE-seq, CIRCLE-seq, and duplex sequencing provide sensitive detection of indels and cryptic promoter activation. Duplex sequencing, with error rates < 10^−7^, enables confident distinction between true off-target edits and polymerase noise, particularly valuable in cardiomyocyte populations where low-frequency variants may affect electrophysiological stability. Long-read sequencing (PacBio HiFi, ONT Q20+) allows structural variant detection, ensuring CRISPRa delivery vectors do not integrate or induce rearrangements—risks highlighted in previous stem cell-based therapies (e.g., insertional oncogenesis from lentiviral platforms).

#### 8.1.2. Mitochondrial Off-Target Analysis

For mtDNA editors, the challenges are distinct. Mitochondrial base editors such as DdCBE and mitoTALENs have negligible indel formation but may cause unintended deamination at sequence-adjacent cytosines. Precision mapping requires full-length mtDNA long-read sequencing, high-fidelity duplex mtDNA sequencing, and mtDNA haplotype tracking to detect low-abundance variants (<0.1% heteroplasmy). This is critical because even small shifts in heteroplasmy can perturb ETC assembly, ATP production, and ROS dynamics [86,87]. High-depth (>20,000×) mitochondrial sequencing is now considered the minimal standard for preclinical mtDNA editing analysis [86,87,88,89]. These risks are consistent with recent mitochondrial engineering work showing that editor delivery, mtDNA packaging constraints, and cytosine-context biases collectively influence editing fidelity and long-term mitochondrial function [90].

Additional profiling using mitochondrial RNA-seq can identify unintended effects on mitochondrial transcriptional units, while mitoproteomic mass spectrometry detects aberrant incorporation of ETC subunits or misfolded proteins. Integrated nuclear–mitochondrial off-target assessment is particularly important in iPSC-CMs, where incomplete mitonuclear coupling already predisposes cells to metabolic fragility.

### 8.2. Immune Responses, Metabolic Dysregulation, and Mitochondrial-Specific Toxicity

#### 8.2.1. Immunogenicity of Delivery Vectors

AAV vectors, despite favorable cardiac tropism, can elicit capsid-specific neutralizing antibodies and T-cell-mediated clearance. Non-viral platforms—lipid nanoparticles (LNPs), polymeric carriers, and synthetic mRNA—reduce genomic integration risk but may activate innate sensors such as TLR7/8 or RIG-I. Repeated dosing amplifies these responses, necessitating immune-compatible formulations and transient expression profiles. iPSC-CMs transplanted into myocardial tissue may encounter inflammatory microenvironments that exacerbate immune activation, highlighting the need for preclinical immune–editing interaction studies.

#### 8.2.2. Risk of Heteroplasmy Shifts

One of the most specific safety risks in mtDNA engineering is heteroplasmy drift. Even on-target editing that selectively cleaves mutant genomes can produce unintended expansion of pre-existing subclones, particularly when combined with metabolic stressors. Sudden shifts in heteroplasmy can alter ETC efficiency, ATP supply, ROS generation, and contractile stability. mtDNA bottleneck effects, observed during iPSC reprogramming [7,8,16], further amplify this vulnerability. Longitudinal single-cell mtDNA sequencing is therefore essential to ensure stable heteroplasmic distributions across differentiation and maturation.

#### 8.2.3. Metabolic Dysregulation and ROS Toxicity

Increasing mitochondrial biogenesis or OXPHOS through PGC-1α–*NRF1/TFAM* activation may overshoot metabolic demand, generating excessive ROS, mitochondrial membrane hyperpolarization, and apoptosis. Studies in engineered cardiac tissues demonstrate that supraphysiological OXPHOS induction destabilizes calcium handling, increases arrhythmogenic susceptibility, and burdens NAD+/NADH cycling [66]. CRISPR-based interventions must therefore be titrated using inducible systems that avoid metabolic overactivation. Multi-omics profiling of redox state, antioxidant capacity, and ETC complex stoichiometry is required to confirm that edited iPSC-CMs achieve balanced bioenergetic function.

#### 8.2.4. Tumorigenicity and Genomic Stability

Although cardiomyocytes exhibit low proliferative capacity, iPSC-derived populations may contain progenitors with residual oncogenic potential. Off-target nuclear activation of growth pathways (e.g., MYC, TGFβ signaling) or insertional mutagenesis from viral vectors can increase tumorigenicity risk. Long-term in vivo studies in immunodeficient mice—extending ≥6–12 months—are the regulatory standard for evaluating proliferation, fibrotic encapsulation, and ectopic tissue formation.

### 8.3. Translational and Regulatory Considerations

The regulatory landscape for mitochondrial genome editing and stem cell-derived cardiomyocyte therapies remains in rapid evolution. Unlike nuclear gene editing, mtDNA editing lacks a defined FDA/EMA pathway, partly due to its non-Mendelian inheritance, heteroplasmy dynamics, and absence of endogenous mitochondrial DNA repair systems. The FDA currently evaluates mtDNA editing technologies under advanced therapy medicinal products (ATMP) classification, requiring:

- Comprehensive off-target and heteroplasmy assessment using validated high-depth sequencing;
- Demonstration of metabolic stability, including OCR, SRC, ROS, and ETC stoichiometry;
- Vector biodistribution and clearance profiles, especially for AAV or replicating non-viral systems;
- Long-term arrhythmogenic and electrophysiological safety, given iPSC-CM susceptibility to early afterdepolarizations;
- Comparability protocols for iPSC lines, differentiation batches, and genome-editing reproducibility.

For nuclear CRISPRa/i therapies, regulators emphasize transcriptional specificity, absence of nuclease activity, and reversibility. Synthetic mRNA- and LNP-based CRISPRa vectors are viewed favorably due to transient expression and lower genotoxicity risks.

Translational success will require biomanufacturing platforms capable of producing GMP-grade edited iPSC-CMs, standardized mitochondrial maturation assays, and harmonized multi-omics release criteria. Integration of CRISPR editing with 3D engineered heart tissues or patch grafts must also meet structural, electrophysiological, and biodistribution standards before progressing to first-in-human trials.

### 8.4. Integrative Perspective

Safety in mitochondrial biogenesis engineering is not merely the absence of toxicity—it is the controlled establishment of mitonuclear harmony. The dual-genome nature of cardiomyocyte energetics demands that editing strategies be precise, transient, and quantitatively validated. High-fidelity sequencing, immune-safe delivery systems, metabolic buffering, and regulatory-aligned manufacturing pipelines form the cornerstone of safe translation. Approached through this rigorously analytical yet forward-looking framework, CRISPR-guided mitochondrial biogenesis can advance toward clinical-grade maturation of iPSC-CMs, opening new trajectories for disease modeling, cardiotoxicity screening, and regenerative cardiology.

Taken together, the safety and translational landscape of mitochondrial biogenesis engineering underscores the need for dual-genome precision, quantitative validation, and immune-compatible delivery systems. Robust off-target profiling, metabolic buffering, and regulatory-aligned workflows are essential to ensure clinical-grade iPSC-CM maturation. These considerations inform the standardized reporting and dataset structures proposed in Section 9 to harmonize methodology across laboratories.

## 9. Standardized Reporting Checklist & Recommended Tables

To harmonize methodological transparency across laboratories and facilitate cross-study comparability, we propose a standardized reporting checklist—emphasizing precision, replicability, and open-science interoperability. A minimal dataset is essential to ensure that mitochondrial biogenesis engineering in iPSC-CMs is evaluable through unified criteria spanning genome editing, metabolic maturation, structural organization, and functional performance. Below, we outline a structured minimal dataset designed to ensure reproducibility across mitochondrial biogenesis engineering studies in iPSC-CMs.

### 9.1. Minimal Dataset for Mitochondrial Biogenesis Engineering Studies

At minimum, all studies should report:

1. Genome Editing Parameters
  - gRNA sequences, PAM sites, promoter targets (PGC-1α, *NRF1/2, TFAM*, *POLG*);
  - Editor type (CRISPRa, CRISPRi, DdCBE, mitoTALENs) and vector/delivery system;
  - On-target efficiency and off-target screening dataset;
  - Editing kinetics (time–dose curves; persistence across passages);
  - Explicit quantitative editing efficiency metrics (e.g., % edited alleles, fold-activation for CRISPRa, DdCBE C•G→T•A conversion rates, mitochondrial editing load per cell);
  - Expanded off-target analysis criteria, including in silico predictions, GUIDE-seq/CHANGE-seq datasets, and mtDNA-wide error-rate profiling where applicable.
2. mtDNA Copy Number & Integrity
  - Absolute mtDNA:nDNA ratio (qPCR/dPCR);
  - Heteroplasmy levels and mutation spectra;
  - Nucleoid size/abundance (TFAM staining, super-resolution imaging).
3. Bioenergetic Profiling (Seahorse/XF)
  - Basal and maximal OCR;
  - Spare respiratory capacity (SRC);
  - ATP-linked respiration;
  - Fatty-acid oxidation (FAO) flux using palmitate-BSA challenge;
  - Proton leak and coupling efficiency.
4. Metabolic Substrate Utilization
  - Glycolytic dependency index;
  - FAO vs. glycolysis switch metrics;
  - NAD+/NADH redox status.
5. Sarcomere & Ultrastructural Organization
  - α-actinin Z-line alignment index;
  - Mitochondria–sarcomere spatial coupling (TEM, confocal, Cryo-ET);
  - Cristae density/lamellar organization (quantitative EM).
6. Electrophysiology
  - Resting membrane potential;
  - Upstroke velocity; APD30/50/90;
  - Calcium transient amplitude, τ decay, SR load.
7. In Vivo or Engineered-Tissue Graft Function
  - Engraftment efficiency;
  - Mitochondrial integration (mitochondrial fusion markers);
  - Host-graft metabolic coupling indices;
  - Arrhythmogenicity metrics and conduction velocity.

### 9.2. Recommended Table Templates

#### 9.2.1. Table Template 1—Tools & Properties

CRISPRa, DdCBE, mitoTALENs

Columns:

- Editor type;
- Molecular mechanism (transcriptional activation, dsDNA nicking, cytosine deamination);
- Target locus (nDNA/mtDNA);
- Editing efficiency metrics (% activation, % conversion, editing load per cell);
- Editing precision & off-target profile;
- Delivery vector compatibility (AAV9, LNP, minicircle DNA, EVs);
- Advantages & limitations;
- Representative studies.

#### 9.2.2. Table Template 2—Maturation Protocols & Outcomes

Rows representing:

- Metabolic conditioning (FAO substrates, glucose restriction);
- Electromechanical stimulation;
- 3D EHT culture;
- CRISPRa-driven PGC-1α/*NRF1/TFAM* upregulation;
- MtDNA editing (DdCBE/mitoTALENs);
- EV-mediated mitochondrial transfer.

Columns:

- Key parameters;
- MtDNA copy number fold-change;
- OCR/SRC improvement (%);
- Cristae maturity index;
- Sarcomere alignment;
- Electrophysiology and Ca^2+^ handling outcomes;
- Off-target/stress response markers;
- Editing efficiency (if applicable to intervention).

#### 9.2.3. Table Template 3—EV/Mitochondrial Transfer Studies

Columns:

- EV isolation method;
- Mitochondrial content quantification;
- Uptake efficiency;
- Impact on OCR/SRC;
- Fusion events with endogenous mitochondria;
- Functional rescue of ETC deficits;
- Scalability and reproducibility considerations.

These harmonized structures serve as a universal blueprint, promoting transparent, reproducible, and inter-laboratory comparable mitochondrial biogenesis research in iPSC-CMs. Together, these minimal datasets and standardized tables ensure that studies of mitochondrial biogenesis capture both genetic and environmental contributions to iPSC-CM maturation. Consistent reporting enables cross-study comparability and informs best-practice workflows, paving the way for next-generation integrative and translational strategies described in Section 10.

## 10. Outstanding Questions & Future Directions

Despite rapid advances in mitochondrial editing and maturation strategies, several unresolved biological and translational challenges must be addressed to enable clinical deployment:

- Mitochondrial Editing & Delivery:

Achieving durable, cell-type-specific correction of mitochondrial genetic defects in cardiomyocytes requires delivery systems that can support high editing efficiency while preserving nucleoid integrity and cellular viability. How can mtDNA delivery be scaled to clinically relevant efficiencies without inducing cytotoxicity or compromising nucleoids? Recent breakthroughs in high-efficiency mtDNA editing suggest that next-generation DdCBE systems can achieve markedly improved on-target specificity and higher editing loads, but their translation to human cardiomyocytes remains untested [71]

- Can next-generation CRISPRa circuits be engineered to be oscillatory, inducible, or self-limiting to prevent hyperproliferation of mitochondria?
- What are optimal vector systems (AAV9, LNP, high-capacity adenoviral, EV-based) for dual nuclear–mitochondrial editing?

Integration with Multi-Omic Benchmarks

- What multi-omic signatures define “adult-equivalent” mitochondrial identity in iPSC-CMs (transcriptome, proteome, metabolome, lipidome, mtDNA epigenome)?
- Can universal functional benchmarks be established linking OCR/SRC, cristae morphology, and Ca^2+^ kinetics to specific omic states?

Biological Fidelity & Line Variability

- How do mitochondrial responses differ across atrial, ventricular, and nodal iPSC-CM subtypes?
- What accounts for inter-line variability in mitochondrial resilience and editing efficiency?

Environmental Synergy

- What sequence and timing of genetic and biophysical interventions best recapitulate postnatal mitochondrial maturation trajectories in iPSC-derived cardiomyocytes?
- Can engineered heart tissues be programmed to self-generate postnatal metabolic transitions?

Translational Roadmap

For mitochondrial maturation strategies to progress beyond proof-of-concept, clear preclinical benchmarks are required to ensure safety, functional durability, and electrophysiological stability.

- What preclinical benchmarks are required to translate:
  ○ Autologous iPSC-CM grafts with corrected mtDNA;
  ○ Ex vivo mtDNA repair for mitochondrial cardiomyopathies;
  ○ CRISPRa-enhanced iPSC-CMs with stable adult-like metabolic identity?
- How can arrhythmogenicity be minimized in engineered mitochondria-dense tissues?

## 11. Conclusions

Engineering mitochondrial biogenesis in iPSC-CMs represents a pivotal strategy to overcome the persistent metabolic and structural immaturity limiting their scientific and clinical utility. Converging evidence from 2010–2025 supports the centrality of PGC-1α–*NRF1/TFAM* signaling, coordinated mtDNA maintenance, and synchronized mitochondrial dynamics in determining energetic competence. CRISPR-guided tools—CRISPRa for transcriptional induction and DdCBE/mitoTALENs for precise mtDNA editing—offer unprecedented control over these axes, yet remain underutilized in cardiomyocyte maturation.

The most actionable insights emerging from this synthesis include:

1. Dual nuclear–mitochondrial targeting is required to achieve adult-equivalent mitochondrial identity, manifested by sustained OXPHOS capacity, organized cristae architecture, and functional excitation–contraction coupling.
2. Standardized readouts—including mtDNA copy number, OCR/SRC, cristae density, sarcomere coupling, and Ca^2+^ kinetics—should be universally reported.
3. Synergistic frameworks integrating genome editing with metabolic conditioning, electromechanical stimulation, and EV-mediated mitochondrial transfer hold the highest promise for translational maturation.
4. Reproducibility and cross-lab harmonization must be prioritized through minimal datasets, table templates, and reporting checklists.

CRISPR-guided nuclear activation forms the primary scaffold for mitochondrial maturation in iPSC-CMs, with mtDNA editing and environmental conditioning acting synergistically to achieve adult-like structural and functional competency.

Future studies should empirically evaluate the synergistic efficacy of CRISPRa-mediated transcriptional activation with environmental conditioning, providing quantitative benchmarks to guide reproducible and translatable mitochondrial maturation protocols.

Collectively, these recommendations delineate a focused roadmap for advancing mitochondrial biogenesis engineering toward clinically viable iPSC-derived cardiomyocytes.

## 12. Supplementary Material Suggestions

To promote open-science reproducibility and facilitate method transfer, we recommend the following.

### 12.1. Extended Data Tables

- Comprehensive lists of gRNAs, PAM sites, and promoter coordinates used in CRISPRa/CRISPRi designs;
- Full mtDNA heteroplasmy tables per sample;
- Quantitative cristae density measurements;
- Metadata for Seahorse experiments (cell counts, normalization factors, substrate conditions).

### 12.2. Extended Methods

- Detailed instructions for:
  ○ CRISPRa activation of PGC-1α/NRF1/TFAM;
  ○ DdCBE and mitoTALEN design, assembly, and validation;
  ○ EV isolation and mitochondrial cargo quantification;
  ○ Metabolic switch protocols (glucose→FAO transition).

### 12.3. Computational Scripts

- Python/R scripts for heteroplasmy quantification;
- mtDNA variant calling pipelines;
- OCR/SRC curve-fitting algorithms;
- Multi-omics integration workflows (PCA, O2PLS, WGCNA).

### 12.4. Sequencing Guidelines

- Minimum sequencing depth for mtDNA amplicons;
- Standards for long-read vs. short-read mtDNA analysis;
- Recommended QC metrics (coverage uniformity, strand bias, error correction models).

These items are proposed as recommended reporting standards for future experimental studies; no supplementary datasets were generated as part of this review

## Data Availability

No new data were created or analyzed in this study. Data sharing is not applicable.
